# The construction, expression, and enhanced anti-tumor activity of YM101: a bispecific antibody simultaneously targeting TGF-β and PD-L1

**DOI:** 10.1186/s13045-021-01045-x

**Published:** 2021-02-16

**Authors:** Ming Yi, Jing Zhang, Anping Li, Mengke Niu, Yongxiang Yan, Ying Jiao, Suxia Luo, Pengfei Zhou, Kongming Wu

**Affiliations:** 1 Department of Oncology, Tongji Hospital of Tongji Medical College, Huazhong University of Science and Technology, 1095 Jiefang Avenue, Wuhan, 430030 People’s Republic of China; 2 Wuhan YZY Biopharma Co., Ltd, Biolake, C2-1, No.666 Gaoxin Road, Wuhan, 430075 People’s Republic of China; 3 Department of Medical Oncology, The Affiliated Cancer Hospital of Zhengzhou University and Henan Cancer Hospital, Zhengzhou, 450008 People’s Republic of China

**Keywords:** Cancer immunotherapy, PD-1, PD-L1, TGF-β, The tumor microenvironment, Bispecific antibody, Immune checkpoint, Immune normalization

## Abstract

**Background:**

Therapeutic antibodies targeting programmed cell death protein 1 (PD-1)/programmed death-ligand 1 (PD-L1) axis induce potent and durable anti-tumor responses in multiple types of cancers. However, only a subset of patients benefits from anti-PD-1/PD-L1 therapies. As a negative regulator of anti-tumor immunity, TGF-β impairs the efficacy of anti-PD-1/PD-L1 and induces drug resistance. Developing a novel treatment strategy to simultaneously block PD-1/PD-L1 and TGF-β would be valuable to enhance the effect of anti-PD-1/PD-L1 and relieve drug resistance.

**Methods:**

Based on the Check-BODY™ technology platform, we developed an anti-TGF-β/PD-L1 bispecific antibody YM101. The bioactivity of the anti-TGF-β moiety was determined by Smad-luciferase reporter assay, transwell assay, western blotting, CCK-8, and flow cytometry. The bioactivity of the anti-PD-L1 moiety was measured by T cell activation assays. EMT-6, CT26, and 3LL tumor models were used to investigate the anti-tumor activity of YM101 in vivo. RNA-seq, immunohistochemical staining, and flow cytometry were utilized to analyze the effect of YM101 on the tumor microenvironment.

**Results:**

YM101 could bind to TGF-β and PD-L1 specifically. In vitro experiments showed that YM101 effectively counteracted the biological effects of TGF-β and PD-1/PD-L1 pathway, including activating Smad signaling, inducing epithelial-mesenchymal transition, and immunosuppression. Besides, in vivo experiments indicated the anti-tumor activity of YM101 was superior to anti-TGF-β and anti-PD-L1 monotherapies. Mechanistically, YM101 promoted the formation of ‘hot tumor’: increasing the numbers of tumor infiltrating lymphocytes and dendritic cells, elevating the ratio of M1/M2, and enhancing cytokine production in T cells. This normalized tumor immune microenvironment and enhanced anti-tumor immune response might contribute to the robust anti-tumor effect of YM101.

**Conclusion:**

Our results demonstrated that YM101 could simultaneously block TGF-β and PD-L1 pathways and had a superior anti-tumor effect compared to the monotherapies.

## Background

It has been well-established that cancer cells could escape from immune surveillance by activating some immune checkpoint pathways [1]. Among all immune checkpoints, programmed cell death protein 1 (PD-1) has attracted most attentions up to now. This cell surface receptor is usually transiently expressed on T cells during priming and expansion [2]. PD-1 has two ligands PD-L1 and PD-L2. Multiple types of cells express PD-L1, including cancer cells and cytokines-stimulated immune cells [3]. In contrast, PD-L2 is mainly expressed on dendritic cells in normal tissues [1, 4]. The binding of PD-1 to PD-L1 or PD-L2 inhibits the activities of T cells. The PD-1-PD-L1 axis is not only an important feedback loop of immune homeostasis but also participates in tumor immune evasion [5, 6].

A series of clinical studies showed that anti-PD-1/PD-L1 antibodies had robust and durable anti-cancer activities across several solid and hematologic cancers, such as lung cancer [7–9], renal cell cancer [10], melanoma [11], hepatocellular carcinoma [12], as well as lymphoma [13–15]. Besides, synergistic anti-tumor responses have been observed in combination of anti-PD1/PD-L1 with PARP inhibition [16] or radiotherapy [17]. Although considerable success has been made in clinic trials, just a subset of patients could benefit from anti-PD-1/PD-L1 treatment, and the overall response rate is relatively low [18, 19]. Actually, for these non-responders undergoing treatment, the PD-1-PD-L1 axis is not the sole speed-limiting step in the Cancer-Immunity Cycle [20]. A group of factors including other immune checkpoints [21–23], cancer neoantigens [24–26], gut microbiota [27, 28], soluble MHC related molecules [29], and cytokines in the tumor microenvironment (TME) also affect anti-cancer immune response [30, 31].

Transforming growth factor-beta (TGF-β) has three isoforms: TGF-β1, TGF-β2, and TGF-β3. As a versatile cytokine, TGF-β is usually overexpressed in advanced tumors and related to poor prognoses [32]. The role of TGF-β is context-dependent. For pre-malignant cells, TGF-β acts as a tumor suppressor by inhibiting cell proliferation, inducing cell apoptosis, and suppressing inflammation [33]. However, for advanced cancers, TGF-β promotes distant metastasis [34], drug resistance [35], and immune escape [36]. TGF-β could regulate the functions of multiple immune cells, such as reducing the cytotoxicity of T cells and natural killer cells (NKs), inducing the differentiation of regulatory T cells (Tregs), and suppressing the antigen presentation of dendritic cells (DCs) [37–40]. Besides, TGF-β restricts the infiltration of immune cells by facilitating the peritumoral collagen generation [30].

In the TME with hyperactive TGF-β signaling, the effect of anti-PD-1/PD-L1 therapy is limited [41]. After anti-PD-1/PD-L1 treatments, the *TGFB1* gene expression is higher in the non-responder’s tumor tissues [30]. Correspondingly, the dual blockade of PD-1/PD-L1 and TGF-β has a synergistic anti-tumor activity [42, 43]. Given that the immunosuppressive effects of the PD-1/PD-L1 axis and TGF-β are independent and complementary, it is rational to block the TGF-β signal to enhance the efficacy of anti-PD-1/PD-L1 and overcome treatment resistance [44]. To optimize the anti-tumor activity of anti-PD-1/PD-L1 therapies, we developed an anti-TGF-β/PD-L1 bispecific antibody YM101, which could simultaneously block the PD-1/PD-L1 and TGF-β pathways.

Check-BODY™ platform is designed by Wuhan YZY Biopharma Co., Ltd for the development of symmetric tetravalency bispecific antibodies. Check-BODY™ platform is characterized by high production yield, easy purification, and high structural stability. YM101 is constructed based on the Check-BODY™ technology platform. In the present study, we explored the biochemistry characteristics of YM101 in vitro and assessed its anti-tumor activity in vivo.

## Materials and methods

### Cell lines and antibodies

CT26 (murine colon cancer cell), EMT-6 (murine breast cancer cell), 4T1 (murine breast cancer cell), A549 (human lung cancer cell), and NCI-H358 (human lung cancer cell) were cultured in RPMI-1640 (Gibco) containing 10% fetal bovine serum (FBS) (Biological Industries). HT-2 (murine T cell) and CTLL-2 (murine T cell) were cultured in RPMI-1640 (ATCC modification, containing glutathione and vitamins) (A10491-01, Gibco) with 10% FBS and 200 IU/ml interleukin-2 (IL-2, Beijing Fourrings). Primary murine T cells were isolated from C57BL/6 mouse-derived splenocytes and cultured in RPMI-1640 containing 10% FBS. NF639 (murine breast cancer cell) and 3LL (murine lung cancer cell) were cultured in DMEM (Gibco) with 10% FBS.

The therapeutic antibodies and isotype control antibody used in the present study included YM101, human IgG, anti-TGF-β, and anti-PD-L1. The anti-TGF-β antibody was constructed based on GC1008 [45]. The anti-PD-L1 antibody was constructed based on the sequence of a chicken anti-PD-L1 single chain variable fragments (scFv) (developed by Jeremy et al.) [46]. All therapeutic antibodies and the human IgG were provided by Wuhan YZY Biopharma Co., Ltd.

### Reduced and non-reduced sodium dodecyl sulfate–polyacrylamide gel electrophoresis (SDS-PAGE)

The prepared YM101 was analyzed using SDS-PAGE and Coomassie Brilliant Blue staining. To verify the purity and molecular weight of YM101, reduced and non-reduced SDS-PAGE were conducted as previously described [47]. After Coomassie Brilliant Blue staining and decolorization, the images of the SDS-PAGE gels were captured with ChemiDoc MP Imaging system (Bio-Rad).

### Capillary electrophoresis with sodium dodecylsulfate

Capillary electrophoresis with sodium dodecylsulfate (CE-SDS) assay was performed following the standard protocol [48]. For the non-reduced CE-SDS, 200 μg sample was mixed with 5 μl Iodoacetamide (0.5 M) and 1 μl 10 KD Internal Standard. After incubation at room temperature for 30 min, the prepared mixture was diluted with SDS-MW buffer (0.05% Tris–HCl, 1% SDS) to 101 μl. Then, the complex was incubated at 60 ℃ for 5 min. For the reduced CE-SDS, 200 μg sample was mixed with 1 μl 10 KD Internal Standard and 5 μl β-mercaptoethanol. The mixture was diluted with SDS-MW buffer to 101 μl. Afterwards, the complex was incubated at 70 ℃ for 5 min. All CE-SDS separations were performed using Beckman PA 800 plus system. UV detection of migrating proteins was detected at 214 nm.

### Measuring molecular weight by liquid chromatograph-mass spectrometer

The molecular weight of the intact antibody was measured with 1 μg/μl YM101, and the molecular weights of the short chain and long chain were measured using reduced samples with Dithiothreitol (50 mM). Liquid chromatograph (LC-30AD, Shimadzu), chromatographic column (MAbPac™ RP, Thermo Fisher), and mass spectrometer (Q Exactive HF-X, Thermo Fisher) were used in this assay.

### Enzyme-linked immunosorbent assays (ELISAs)

96 well flat-bottom plates (9018, Corning) were coated with TGF-β1 (Z03411, Genscript), TGF-β2 (Z03429, Genscript), TGF-β3 (Z03430, Genscript), and PD-L1 (50010-M03H, Sino Biological) (200 ng per well) at 4 °C overnight. On the next day, the plates were washed 3 times using PBS containing 0.05% Tween-20 (30189328, Sinopharm Chemical Reagent) (PBST). The assay plates were blocked with PBS containing 3% bovine serum albumin (BSAS 1.0, BOVOGEN) for 2 h. Then, serially diluted YM101 or controls were added into the plates and incubated for 1 h. Afterwards, the plates were washed and incubated with anti-hIgG-HRP (1:5000, A80-319P, Bethyl) for 1 h. ELISA substrates (100 μl per well) (555214, BD Biosciences) were added into plates, and the HRP reaction was terminated using 2 N HCl. The absorbance values were read at 450 nm (Molecular Devices) [49].

For the double-antigen sandwich ELISA assay, 96 well plates (9018, Corning) were coated with TGF-β1, TGF-β2, and TGF-β3 (200 ng per well) at 4 °C overnight. After washing and blocking, serially diluted YM101 or controls were added into the plates and incubated at 37 °C for 1 h. Then, the plates were washed and incubated with PD-L1-Biotin (200 ng/ml, 100 μl per well; murine PD-L1, 50010-M03H, Sino Biological; Biotin labeling Kit-NH2, LK03, Dojindo; Biotin labeling was performed according to the recommendations of manufacturers) for 1 h. Subsequently, the assay plates were washed and incubated with peroxidase-conjugated streptavidin (1:5000, SA00001-0, Proteintech). According to the standard protocol of ELISA, the simultaneous binding of YM101 to TGF-β and PD-L1 was quantified by absorbance value.

### Smad luciferase reporter assay

3 × 10^4^ viable NF639 or 4T1 cells were seeded in 96 well flat-bottom plates (3904, Corning) and incubated at 37 ℃ overnight. NF639 or 4T1 cells were transiently transfected with Smad luciferase reporter plasmid (0.1 μg per well) by Effectene® Transfection Reagent (301425, QIAGEN). After transfection, NF639 or 4T1 cells were incubated with TGF-β1 (10 ng/ml) and serially diluted YM101 or controls for 24 h. Luminescence was detected using Bright-Glo™ Luciferase Assay System (E2620, Promega).

### Transwell migration and invasion assay

Transwell migration or invasion assays were performed using 8.0 µm pore size inserts (3422, Corning) without or with Matrigel (356234, BD Biosciences). NF639 and 4T1 cells were treated with 10 ng/ml TGF-β1 plus 10^5^ pM antibodies for 96 h. Untreated cells were employed as the negative control. All cells were cultured with DMEM or RPMI-1640 containing 1% FBS during treatment. Then, 5 × 10^4^ NF639 and 4T1 cells were suspended in 100 µl DMEM or RPMI-1640 containing 1% FBS and seeded in the upper chambers. The lower chambers were filled with 600 µl DMEM or RPMI-1640 containing 10% FBS. Migratory and invasive cells were stained with crystal violet solution after incubating for 12 h.

### Western blotting

Protein was extracted by Mammalian Total Protein Extraction Kit (DE101-01, Transgen). 25 µg protein from each sample was separated by SDS-PAGE gel and then transferred to a polyvinylidene fluoride membrane (Millipore). The primary antibodies were anti-E-cadherin (1:1000, 3195, CST), anti-N-cadherin (1:1000, 13116, CST), anti-Vimentin (1:1000, 5741, CST), anti-Snail (1:1000, 3879, CST), anti-β-Actin (1:1000, 8457, CST), anti-phospho-Erk1/2 (1:1000, 4370, CST), anti-Erk1/2 (1:1000, 9102, CST), and anti-GAPDH (1:1000, 5174, CST). The secondary antibodies were anti-rabbit-IgG-HRP (1:2000, 7074, CST). Signal detection was performed as previously described [50].

### Treg induction assay

96 well flat-bottom plates (3599, Corning) were pre-coated with 5 μg/ml anti-CD3 (100302, BioLegend) at 4 °C overnight. After lysing red blood cells (C3702-120 ml, Beyotime), murine splenocytes were washed and cultured in RPMI-1640 containing 10% FBS, 2 μg/ml anti-CD28 (102116, BioLegend), 10 ng/ml TGF-β1, 100 IU/ml IL-2 for 6 days. Fixable Viability Stain 700 (564997, BD Biosciences), anti-CD4 (100510, BioLegend), anti-CD25 (102038, BioLegend), anti-Foxp3 (126404, BioLegend), eBioscience™ FOXP3/Transcription Factor Staining Buffer Set (00-5523-00, Invitrogen) were used to detect the ratio of Treg.

### CCK-8 assay

TGF-β could inhibit the IL-2 dependent proliferation of CTLL-2 and HT-2. We performed CCK-8 assays to measure the capability of YM101 antagonizing the effect of TGF-β. CTLL-2 and HT-2 cells (1 × 10^3^ per well) were seeded in 96-well plates (3599, Corning). Then, TGF-β1 and antibodies (10^5^ pM) were added into plates. Within one week after treatment, cell viability was continuously monitored by CCK-8 reagent (10 μl per well, LK04, Dojindo).

### Cell cycle and apoptosis assays

CTLL-2 and HT-2 were pretreated with TGF-β1 (10 ng/ml) plus antibodies (10^5^ pM) for 4 days. Then, cells were harvested for cell cycle analysis. After treatment with 75% cold ethanol at 4 °C for 30 min, cells were dyed by the staining buffer containing 50 μg/ml Propidium Iodide (PI) (P4170-10MG, Sigma) and 200 μg/ml RNase (GE101-01, Transgen) for 30 min. The ratios of cells in different phases were detected by flow cytometry and analyzed by Flowjo v10 (Ashland, OR).

CTLL-2 and HT-2 were pretreated with TGF-β1 (10 ng/ml) plus antibodies (10^5^ pM) for 5 days. Cell apoptosis was evaluated according to the standard protocol of manufacturers (Annexin V/PI Apoptosis Detection Kit, 640914, BioLegend) [51]. For each test, 1 × 10^5^ cells were suspended with 200 μl Annexin V binding buffer, 5 μl Annexin V, and 10 μl PI. After incubation for 20 min at room temperature, the ratio of apoptotic cells was measured by flow cytometry.

### T cell activation assay

TGF-β regulates the differentiation of naïve T cells and affects the levels of multiple cytokines during T cell activation [52]. In the presence of exogenous TGF-β1, we investigated the YM101-caused alterations in the cytokine pattern. Murine T cells were isolated from C57BL/6 mouse-derived splenocytes by Dynabeads™ Untouched™ Mouse T Cells Kit (11413D, Invitrogen). T cell activation assay was performed using precoated anti-CD3 (2 μg/ml) and anti-CD28 (2 μg/ml). The T cells (1 × 10^6^/ml) were mixed with TGF-β1 (10 ng/ml) and antibodies (10^5^ pM) and cultured at 37 ℃ for 4 days. Then, the cellular supernatants were harvested, and the concentrations of cytokines were measured by Multi-Analyte Flow Assay Kit (741044, MU Th Cytokine Panel, BioLegend).

Additionally, to assess the activity of the anti-PD-L1 moiety of YM101, we performed a T cell activation assay (precoated anti-CD3: 2 μg/ml, anti-CD28: 2 μg/ml) in the presence of exogenous PD-L1 (2 μg/ml). Viable T cells (1 × 10^6^/ml) were mixed with PD-L1 and antibodies (10^5^ pM). The T cells were cultured at 37 °C for 4 days. Then, the cellular supernatants were harvested, and the IL-2 concentration was measured by Multi-Analyte Flow Assay Kit. Besides, we detected the T cell proliferation by CFSE (5 μM, 565082, BD Biosciences) dilution assays. Murine T cells were activated by precoated anti-CD3 (2 μg/ml) and cultured with exogenous PD-L1 (2 μg/ml) as well as antibodies (10^5^ pM) for 4 days. The ratio of daughter cells was measured by flow cytometry.

### Murine tumor models

The anti-tumor effect of YM101 was evaluated in multiple syngeneic tumor models in immunocompetent mice, including EMT-6, CT26, and 3LL.

### Orthotopic EMT-6 model

To explore the optimal dose of YM101, BALB/c mice were inoculated with 5 × 10^4^ EMT-6 cells in the right mammary fat pad. Treatment was initiated 7 days later, when the tumor volume reached ≈ 100 mm^3^. Tumor size was measured with a digital caliper three times a week. Tumor volume was calculated using the following formula: tumor volume (mm^3^) = length × width^2^ × 0.5. Mice were euthanized when tumor volume exceeded 2500 mm^3^ or when the study ended.

To investigate the effect of YM101 on the TME, BALB/c mice were inoculated in the right mammary fat pad with 5 × 10^4^ EMT-6 cells. Therapy was initiated on 11 to 12 days after inoculation, when the tumor volumes reached ≈ 250 mm^3^. Mice were randomly assigned to 4 groups (8 mice per group): vehicle (saline), anti-PD-L1, anti-TGF-β, and YM101. Mice received equivalent mole antibodies every two days by intraperitoneal injection. The mice received treatments for 6 times. Tumor size was measured with a digital caliper every two days.

### Subcutaneous CT26 model

1 × 10^6^ CT26 cells were inoculated subcutaneously in BALB/c mice. Treatment was started on 9 to 10 days after inoculation, when the tumor volumes reached 100–200 mm^3^. Mice received treatment every two days by intraperitoneal injection. Tumor size was measured with a digital caliper every 2 days.

### Subcutaneous 3LL model

C57BL/6 mice were inoculated with 1 × 10^6^ 3LL cells in the right flank. Therapy was initiated on 8 days after inoculation when the tumor volumes reached ≈ 100 mm^3^. Mice received treatment every two days. On the 10 days after the final YM101 treatment, YM101-cured or treatment-naive C57BL/6 mice were rechallenged by subcutaneously inoculating 1 × 10^6^ 3LL cells. Additionally, we repeated the 3LL challenging assay to explore the effect of YM101 on survival. 3LL tumor-bearing mice received 6 doses of antibodies. Then, the mice were followed up for survival for 2 weeks.

### Flow cytometry for immune profiling

Mice were sacrificed, and tumor tissues were harvested. Single-cell suspensions were prepared using Collagenase B (1 mg/ml, 11088807001, Roche) and Hyaluronidase (1 mg/ml, abs47014926, Absin). After digestion, the suspensions were filtered by 40 μm Nylon cell strainers (352340, Corning). Before staining, cells were suspended in PBS and dyed by Fixable Viability Stain 700. Fluorescent staining was performed according to the manufacturer’s recommendations. Fluorescent antibodies recognizing murine CD45 (557659, BD Biosciences), CD3 (100306, BioLegend), CD8a (100722, BioLegend), CD107a (121629, BioLegend), granzyme B (372204, BioLegend), CD11b (101206, BioLegend), I-A/I-E (107608, BioLegend), CD11c (566504, BD Biosciences), CD206 (141708, BioLegend), F4/80 (565411, BD Biosciences) were used in this assay. Flow cytometry buffers utilized in the experiment included Brilliant Stain Buffer (563794, BD Biosciences) and eBioscience™ FOXP3/Transcription Factor Staining Buffer Set (00-5523-00, Invitrogen). Flow cytometry was performed using Beckman CytoFLEX S or Beckman CytoFLEX LX. Flow cytometry data were analyzed by Flowjo v10 (Ashland, OR).

### Picrosirius red staining and other immunohistochemistry assays

Isolated tumors were fixed using 4% paraformaldehyde for 48 h, dehydrated, and embedded with paraffin wax. Tumor tissues were sectioned and transferred to slides. Picrosirius red staining was conducted using Direct Red 80 (365548, Sigma-Aldrich). Anti-TGF-β1 (ab215715, Abcam), anti-p-Smad3 (ab52903, Abcam), anti-α-SMA (AF1032, Affinity Biosciences), anti-E-cadherin (3195, CST), anti-Vimentin (CY5134, Abways), anti-Ki67 (ab16667, Abcam), anti-PCNA (BM0104, Boster), anti-cleaved-Caspase 3 (9664, CST), anti-CD3 (ab16669, Abcam), anti-CD4 (ab183685, Abcam), and anti-CD8 (ab217344, Abcam) immunohistochemistry staining assays were performed according to the two-step protocol [53]. Bright-field images were captured by Hamamatsu Nanozoomer slide-scanning platform. The regions of interest (ROIs) were defined by two pathologists.

The digital quantitation of immunohistochemistry staining was conducted with ImageJ software (National Institutes of Health). The ratio of CD3^+^ cell was assessed using the proportions of positive pixels in ROIs. For picrosirius red, anti-α-SMA, anti-E-cadherin, anti-Vimentin, anti-Ki67, anti-PCNA, and anti-cleaved-Caspase 3 staining, the expression abundances were measured by integral optical density (IOD) values. For any ROI, the infiltration depth of T cell was calculated by the mean nearest distance of all CD3^+^ cells to the tumor border. The mean nearest distance was scaled by the distance between the corresponding tumor border to tumor center [30].

### RNA-seq assay

The total RNA of EMT-6 tumors was extracted by Trizol, as previously described [53]. For each group, randomly selected 4 samples were collected for the RNA-seq assay. Mus_musculus.GRCm38 was used as the reference genome. Differentially expressed genes (DEGs) were analyzed using R software (4.0) with edgeR package. DEG was identified as the gene with fold change over 2 and *p*-value less than 0.05. The comparisons were performed as following: YM101 vs. vehicle, YM101 vs. anti-TGF-β, and YM101 vs. anti-PD-L1. The expression profile of DEGs was visualized using pheatmap package. The immune signatures were designed based on public lists [54], which were summarized in Additional file 1: Table S1. The signature was scored as the mean value of scaled expression levels of all involved genes, and differences were compared by ROAST algorithm [55].

### Statistical analyses

Statistical analyses were conducted using Prism 8 (GraphPad Software Inc.). For data following a normal distribution, Student’s *t*-test or *t*-test with Welch’s correction was used for the statistical comparison between two groups. For data not meeting normal distribution, Mann–Whitney test was used for the statistical analysis between two groups. All tests were two-sided, and the significance level was 0.05.

## Results

### The structure of YM101

**Fig. 1:**
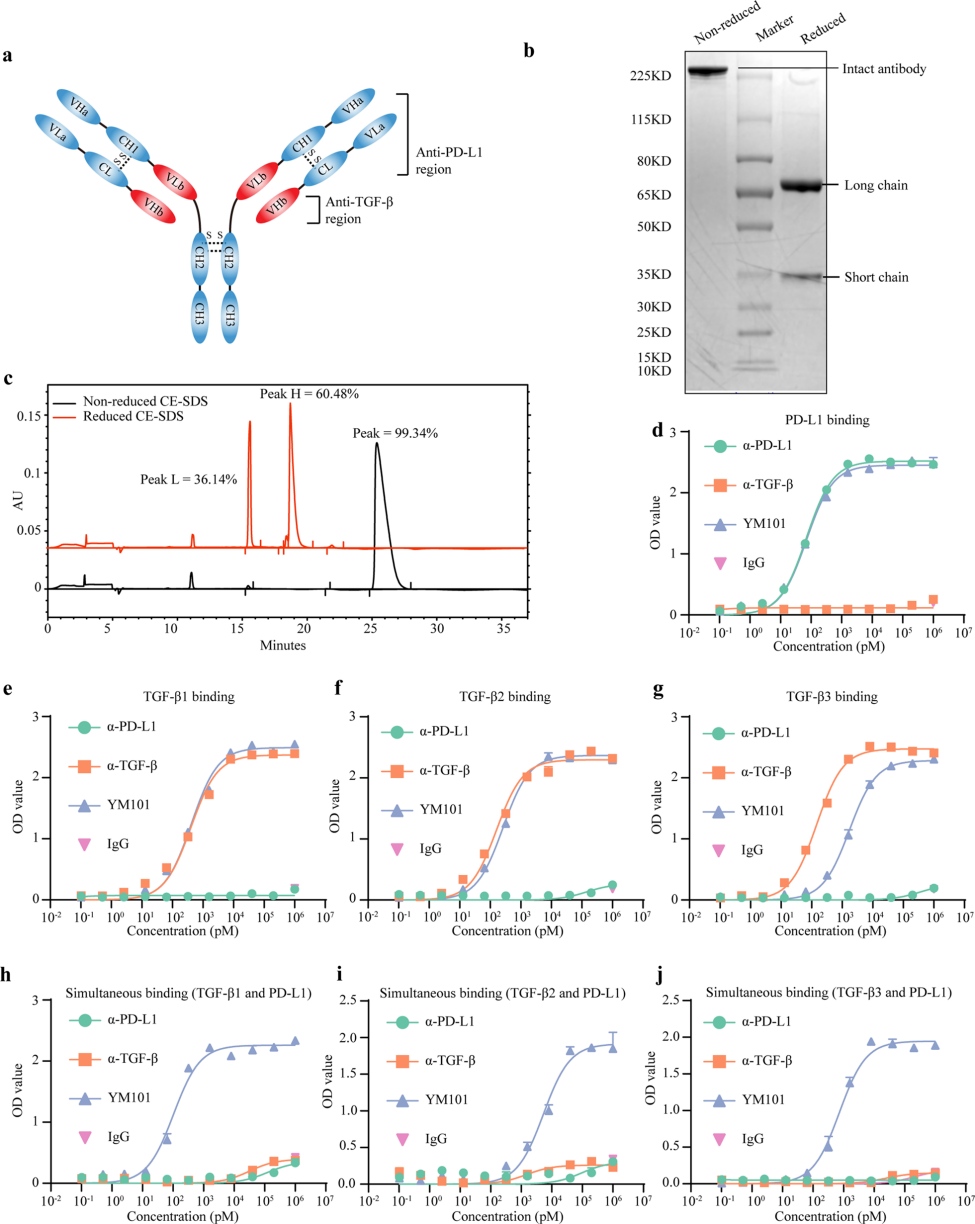
The basic characteristics of YM101. **a** The structure of YM101. YM101 contains two anti-PD-L1 regions and two anti-TGF-β regions. The Fc region of YM101 is an IgG1/IgG2 hybrid fragment: the CH2 is from IgG2, and the CH3 is from IgG1. **b** The results of non-reduced and reduced SDS-PAGE assays. A single band was observed in the lane of non-reduced YM101, and two bands were found in the lane of reduced YM101. **c** The results of non-reduced and reduced CE-SDS assays. In non-reduced CE-SDS, one peak was detected. In reduced CE-SDS, two peaks were detected (one for short chain and the other for long chain). The purity of YM101 is over 99%. **d** The binding of YM101 to PD-L1. Serially diluted YM101 or controls were incubated with plate-coated PD-L1. The binding affinity was measured by anti-hIgG ELISA. **e**–**g** The binding of YM101 to TGF-β. Serially diluted YM101 or controls were incubated with plate-coated TGF-β1, TGF-β2, and TGF-β3. The binding affinity was measured by anti-hIgG ELISA. **h**–**j** The simultaneous binding to TGF-β and PD-L1. Serially diluted YM101 or controls were incubated with plate-coated TGF-β1, TGF-β2, and TGF-β3. Then, PD-L1-Biotin and peroxidase-conjugated streptavidin were used for ELISA assays. α-TGF-β: anti-TGF-β, α-PD-L1: anti-PD-L1

YM101 is a recombinant IgG1/IgG2 hybrid antibody. The short chain of YM101 consists of three domains: VLa, CL, and VHb (Fig. 1a). The VLa is designed based on the anti-PD-L1 scFv (developed by Jeremy et al.) [46]. The VHb domain is identical to the VH domain of the anti-TGF-β antibody. The long chain of YM101 contains five domains: VHa, CH1, VLb, CH2, and CH3. The VHa is designed based on the sequence of anti-PD-L1 scFv, and the VLb is from VL of the anti-TGF-β antibody. The Fc of YM101 is a hybrid fragment with a mutation in D270A. The CH2 domain is derived from IgG2, and the CH3 domain is derived from IgG1.

As indicated in Fig. 1b, a single band was observed in the lane of non-reduced YM101, and two bands were found in the lane of reduced YM101. The purity of prepared YM101 is over 99% in the CE-SDS assay (Fig. 1c). The molecular weight of intact YM101 is about 204.0 KD (36.4 KD for the short chain; 65.5 KD for the long chain) (Additional file 1: Figure S1a–c).

### YM101 specifically bound to PD-L1 and TGF-β

YM101 bound to the precoated PD-L1 with a profile similar to anti-PD-L1 (*K*_d_ = 71 pM for YM101, 70 pM for anti-PD-L1) (Fig. 1d). Relative to anti-TGF-β, YM101 bound to the precoated TGF-β1 and TGF-β2 with similar affinities (TGF-β1: *K*_d_ = 418 pM for YM101, 402 pM for anti-TGF-β; TGF-β2: *K*_d_ = 261 pM for YM101, 161 pM for anti-TGF-β). However, for TGF-β3, the affinity of YM101 was weaker than anti-TGF-β (TGF-β3: *K*_d_ = 1719 pM for YM101, 146 pM for anti-TGF-β) (Fig. 1e–g). In the double-antigen sandwich ELISA assays, YM101 captured by plate-bound TGF-β could simultaneously bind to PD-L1 (TGF-β1: *K*_d_ = 104 pM; TGF-β2: *K*_d_ = 5348 pM; TGF-β3: *K*_d_ = 729 pM) (Fig. 1h–j).

### YM101 inhibited TGF-β-induced Smad signaling and epithelial-mesenchymal transition (EMT)

**Fig. 2:**
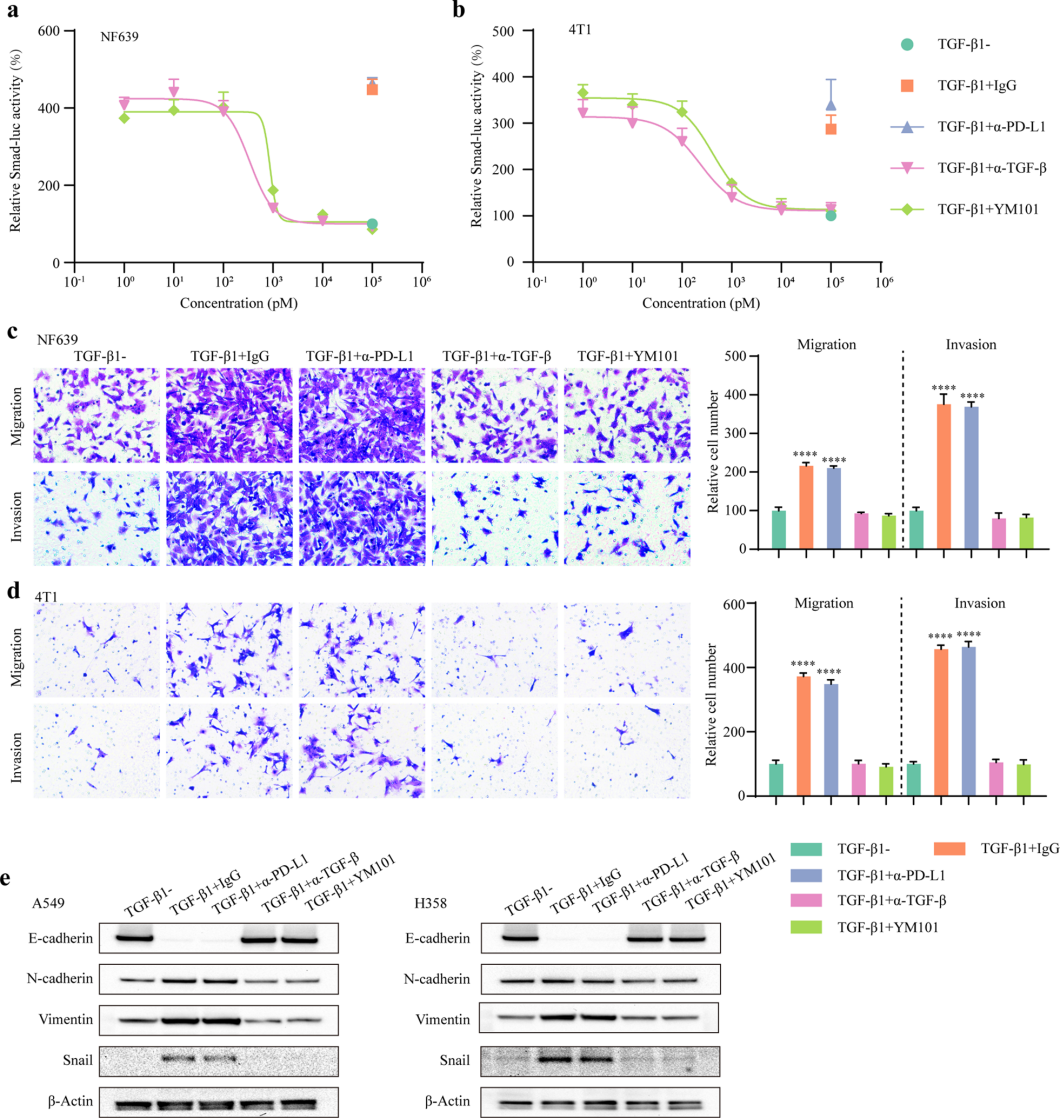
The antagonistic effect of YM101 on TGF-β signaling pathway and epithelial-mesenchymal transition in cancer cells. **a**, **b** Smad luciferase reporter assay to measure the effect of YM101 on canonical TGF-β signaling. In the presence of TGF-β1 (10 ng/ml), Smad-luc-transfected NF639 and 4T1 cells were incubated with YM101 or controls for 24 h. Then, luminescence was detected. **c**, **d** Transwell migration and invasion assays to determine the effect of YM101 on TGF-β-regulated cell movement in cancer cells. **e** Western blotting assays to measure the effect of YM101 on TGF-β-mediated epithelial-mesenchymal transition in cancer cells. After treatment with TGF-β1 (10 ng/ml) plus antibodies for 4 days, epithelial-mesenchymal transition-associated markers were detected. *p < 0.05, **p < 0.01, ***p < 0.001, and ****p < 0.0001 denote the significant difference relative to YM101 treatment. α-TGF-β: anti-TGF-β, α-PD-L1: anti-PD-L1

As the main intercellular effector of TGF-β receptor, Smad proteins could translocate to cell nuclear and regulate transcription [56]. We measured the blocking effect of YM101 on TGF-β pathway by Smad-luciferase reporter assays. The results showed that YM101 inhibited the TGF-β-stimulated Smad transcription activity in NF639 (IC_50_ = 866 pM) and 4T1 (IC_50_ = 417 pM) (Fig. 2a, b).

TGF-β enhances the movement capability and promotes the EMT in cancer cells [57]. Consistent with previous observations, TGF-β1 promoted the migration and invasion of NF639 and 4T1 cells. YM101 abrogated the TGF-β1-enhanced cell movement (Fig. 2c, d). Also, TGF-β1 decreased epithelial marker while increased the expression of mesenchymal markers in A549 and NCI-H358 cells. YM101 effectively antagonized the TGF-β1-induced EMT in A549 and NCI-H358 cells: upregulating epithelial marker (E-cadherin) and downregulating mesenchymal markers (N-cadherin and Vimentin) as well as EMT-associated transcriptional factor (Snail) (Fig. 2e). At the same time, anti-PD-L1 did not affect the EMT in cancer cells. Additionally, we found TGF-β1 activated Smad-independent MAPK pathway. YM101 restored the TGF-β1-induced phosphorylation of Erk (Additional file 1: Fig. 2).

### YM101 reversed the TGF-β-caused immunosuppression

**Fig. 3:**
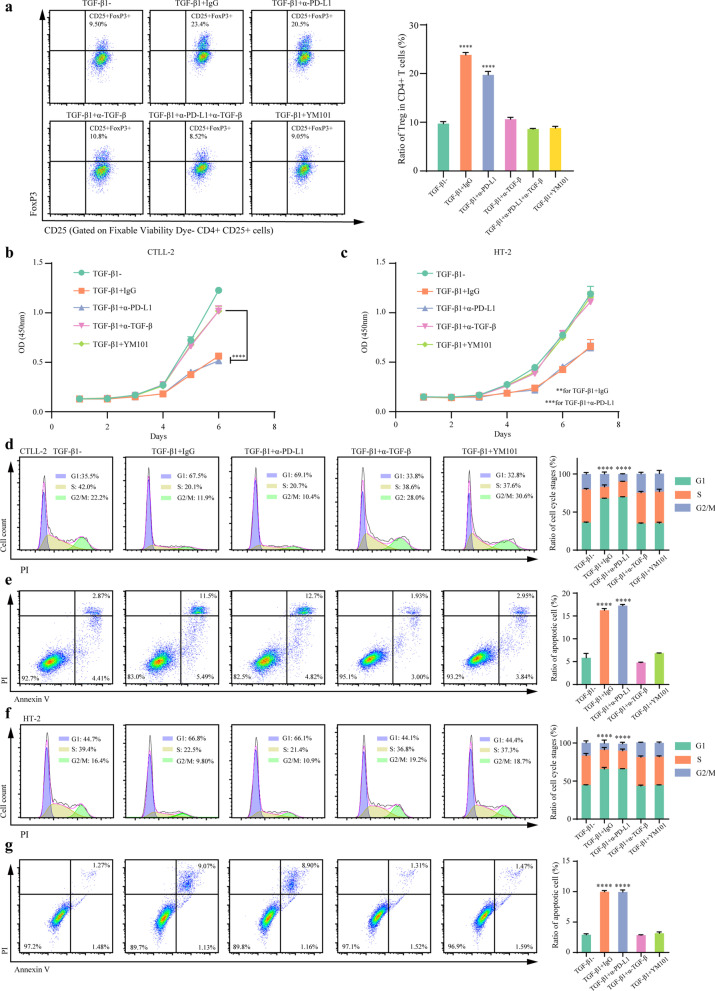
YM101 counteracted TGF-β1-induced the differentiation of Tregs, proliferation inhibition, and apoptosis of T cells. **a** YM101 suppressed the differentiation of Tregs caused by TGF-β1. Murine splenocytes were treated with plated-coated CD3, CD28, IL-2, TGF-β1, and YM101 or controls. The results of flow cytometry showed the ratio of Treg in CD4^+^ T cells. **b**, **c** YM101 reversed the TGF-β1-caused proliferation inhibition in T cells. **d** YM101 counteracted the TGF-β1-caused alterations in cell cycle distribution in CTLL-2. **e** YM101 relieved the TGF-β1-mediated apoptosis in CTLL-2. **f** YM101 reversed the TGF-β1-caused alterations in cell cycle distribution in HT-2. **g** YM101 hedged the TGF-β1-mediated apoptosis in HT-2. *p < 0.05, **p < 0.01, ***p < 0.001, and ****p < 0.0001 denote the significant difference relative to YM101 treatment. α-TGF-β: anti-TGF-β, α-PD-L1: anti-PD-L1

TGF-β cooperates with IL-2 to induce Foxp3 expression and promotes the conversion of naïve T cells to Tregs [58]. Additional TGF-β1 significantly increased the ratio of Tregs in vitro. Unlike IgG and anti-PD-L1, YM101 effectively suppressed the differentiation of Tregs caused by TGF-β1 (*p* < 0.0001) (Fig. 3a). Moreover, for IL-2 dependent murine T cell lines CTLL-2 and HT-2, exogenous TGF-β1 hampered T cell proliferation, increased the ratio of cells in G1 phase, and promoted cell apoptosis. YM101 blocked the negative effects of TGF-β1 on T cells: reversing proliferation inhibition, decreasing the ratio of G1, and counteracting cell apoptosis (Fig. 3b–g).

**Fig. 4:**
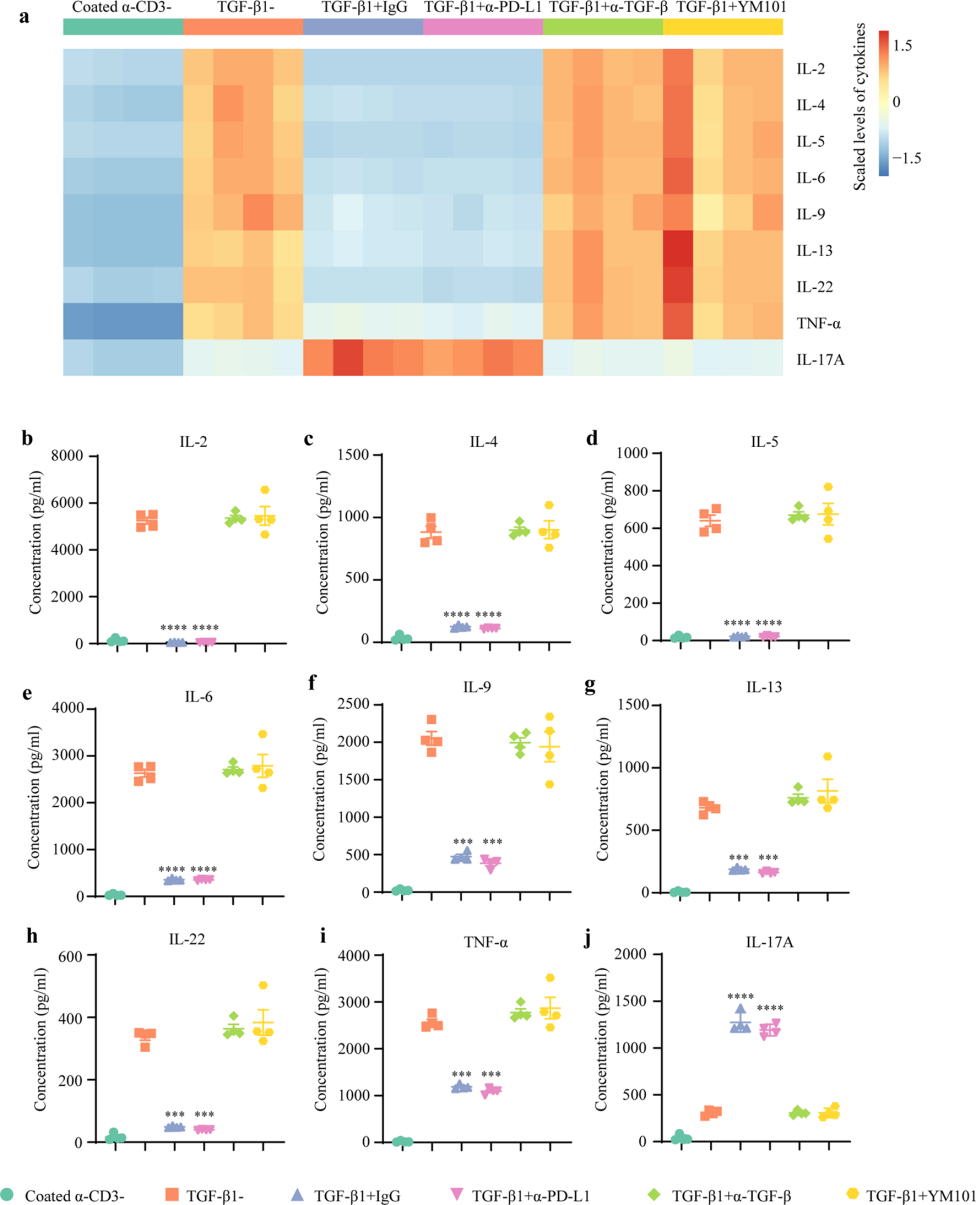
YM101 antagonized the TGF-β1-caused changes in the cytokine release. **a** The heatmap showing the effects of TGF-β1 and YM101 on cytokine release during T cell activation. **b**–**j** YM101 reversed TGF-β1-mediated alterations in cytokine release including IL-2, IL-4, IL-5, IL-6, IL-9, IL-13, IL-22, TNF-α, and IL-17A. *p < 0.05, **p < 0.01, ***p < 0.001, and ****p < 0.0001 denote the significant difference relative to YM101 treatment. α-TGF-β: anti-TGF-β, α-PD-L1: anti-PD-L1

Besides, TGF-β1 substantially reshaped the cytokine pattern during T cell activation (Fig. 4a). Most cytokines, such as Th1-associated (IL-2), Th2-associated (IL-4, IL-5, and IL-13), and pro-inflammatory cytokines (IL-6 and TNF-α), were downregulated by exogeneous TGF-β1. Conversely, the level of IL-17 was increased by exogeneous TGF-β1. YM101 almost completely antagonized the TGF-β1-caused changes in the cytokine release (Fig. 4b–j).

### YM101 suppressed the activity of the PD-1/PD-L1 axis

**Fig. 5:**
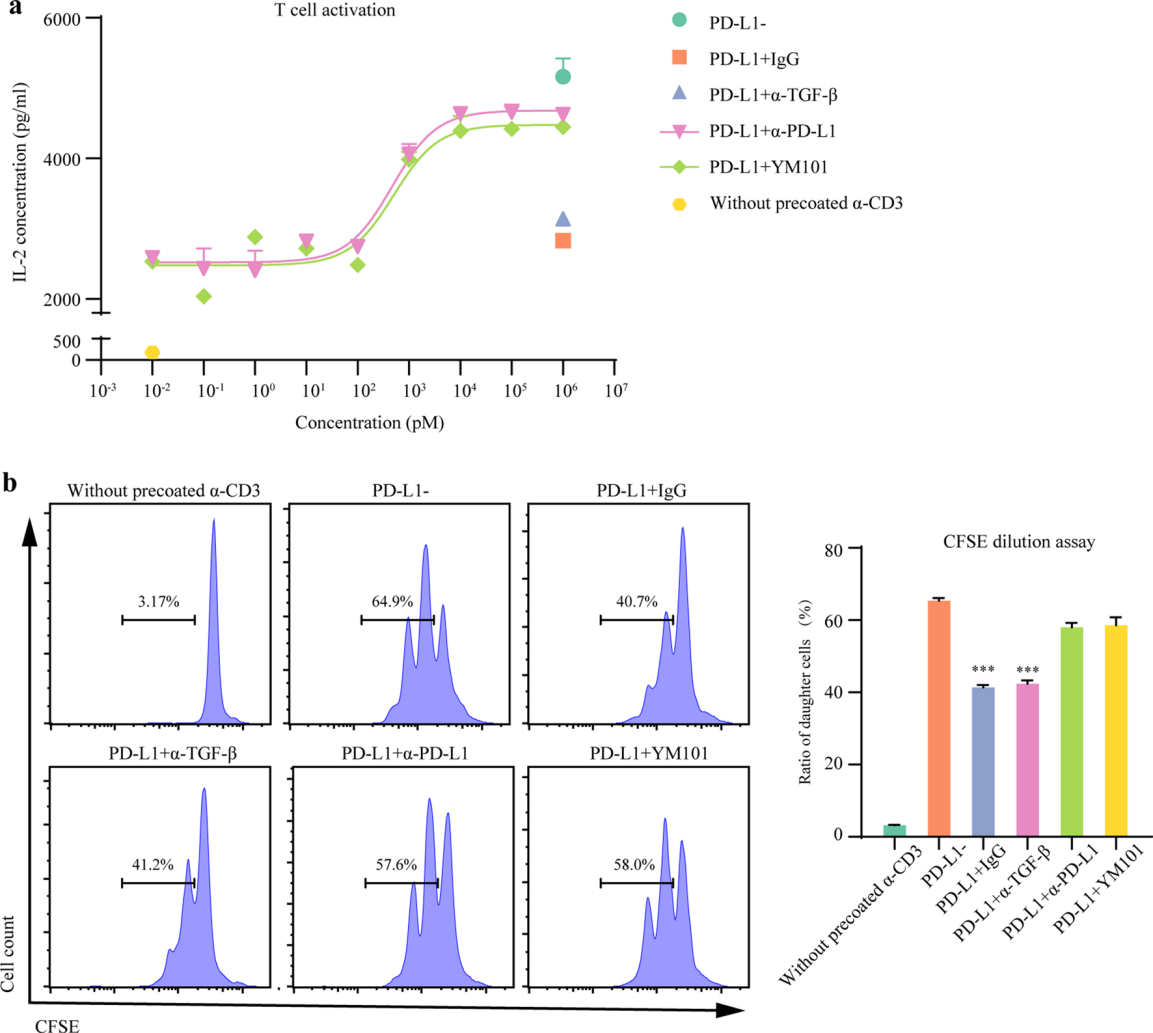
YM101 counteracted the PD-1/PD-L1-mediated immunosuppression. **a** YM101 reversed the PD-1/PD-L1 axis-caused inhibition of IL-2 generation. The T cell activation assay was performed in the presence of exogeneous PD-L1 and YM101 or controls. **b** The CFSE dilution assays showed that YM101 antagonized the PD-1/PD-L1 axis-mediated proliferation inhibition in T cells. *p < 0.05, **p < 0.01, ***p < 0.001, and ****p < 0.0001 denote the significant difference relative to YM101 treatment. α-TGF-β: anti-TGF-β, α-PD-L1: anti-PD-L1

IL-2 is a vital cytokine in T cell activation, which is significantly inhibited by the PD-1/PD-L1 axis [59]. In the presence of exogenous PD-L1, IL-2 secretion was suppressed. YM101 abrogated the PD-L1-mediated downregulation of IL-2 in a dose-dependent manner (EC_50_ = 506.9 pM) (Fig. 5a). Besides, the results of CFSE dilution assay showed that YM101 reversed the PD-1/PD-L1 axis-inhibited proliferation of T cells (Fig. 5b).

### YM101 inhibited tumor growth in murine models

**Fig. 6:**
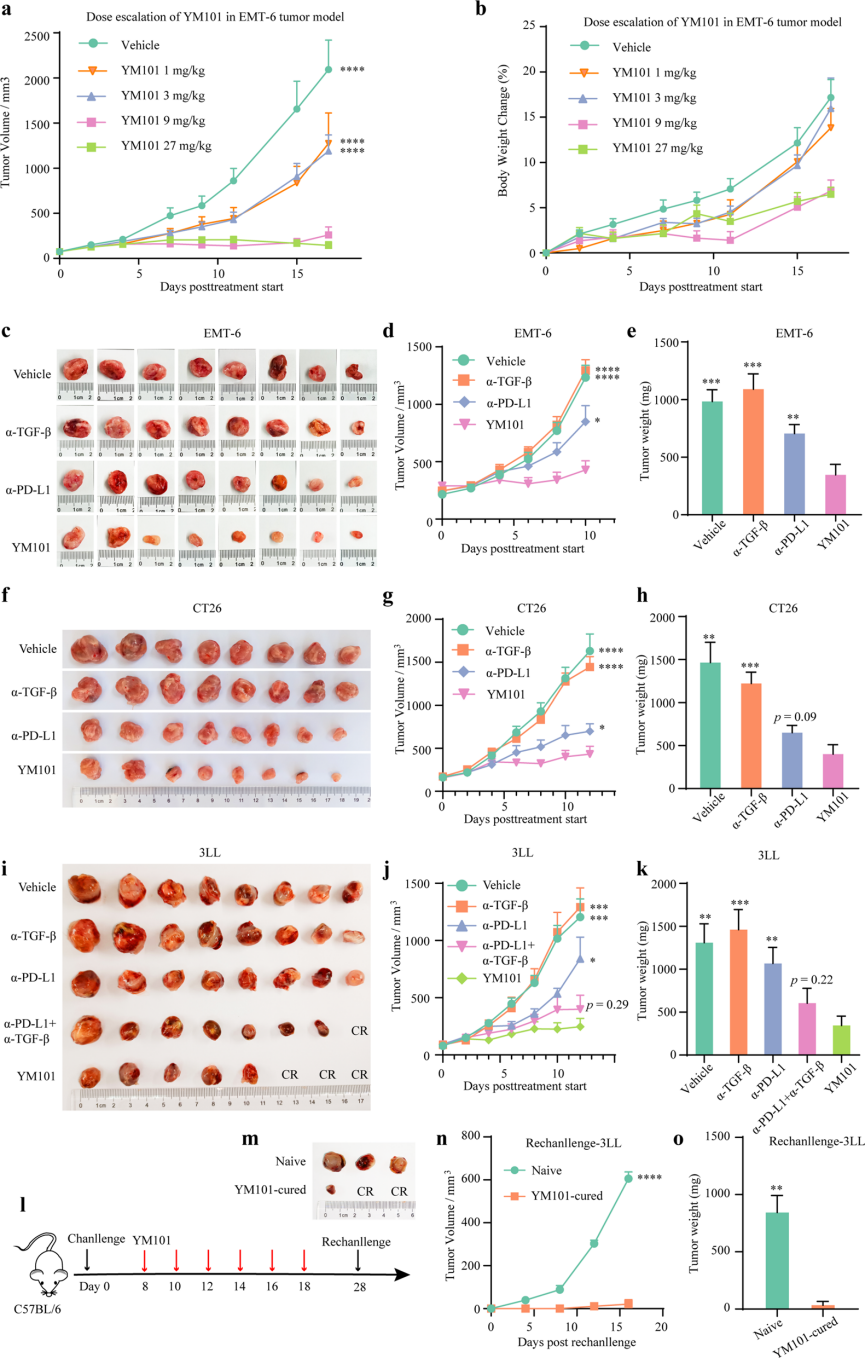
YM101 treatment inhibited tumor growth in murine tumor models. **a** Exploring the optimal dosage of YM101 in the EMT-6 model. The growth curves of EMT-6 tumors of mice receiving different dosages of YM101. **b** The body weight change curves of EMT-6-bearing mice receiving different dosages of YM101. **c** The tumor images of EMT-6-bearing mice receiving the treatment of YM101 or controls. **d** The tumor growth curves of EMT-6-bearing mice receiving the treatment of YM101 or controls. **e** The tumor weights of EMT-6-bearing mice receiving the treatment of YM101 or controls. **f** The tumor images of CT26-bearing mice receiving the treatment of YM101 or controls. **g** The tumor growth curves of CT26-bearing mice receiving the treatment of YM101 or controls. **h** The tumor weights of CT26-bearing mice receiving the treatment of YM101 or controls. **i** The tumor images of 3LL-bearing mice receiving the treatment of YM101 or controls. **j** The tumor growth curves of 3LL-bearing mice receiving the treatment of YM101 or controls. **k** The tumor weights of 3LL-bearing mice receiving the treatment of YM101 or controls. **l** For the rechallenge assay, YM101-cured or treatment-naïve mice were inoculated with 1 × 10^6^ 3LL cells on the day 10 after the final YM101 injection. **m** The tumor images of the 3LL rechallenge assay. **n** The tumor growth curves of the 3LL rechallenge assay. **o** The tumor weights of the 3LL rechallenge assay. *p < 0.05, **p < 0.01, ***p < 0.001, and ****p < 0.0001 denote the significant difference relative to YM101 treatment. α-TGF-β: anti-TGF-β, α-PD-L1: anti-PD-L1, CR: complete regression

Firstly, we explored the anti-tumor effect of different doses of YM101. In the EMT-6 orthotopic tumor model, the low-dose of YM101 (1 mg/kg or 3 mg/kg) had a modest anti-tumor effect (Fig. 6a). At the same time, the middle- (9 mg/kg) and high-dose (27 mg/kg) of YM101 showed a potent anti-tumor activity superior to the low-dose of YM101. Tumor was completely regressed in some mice receiving the middle-dose (3 of 10) and high-dose (3 of 10) of YM101 therapy. However, no complete tumor regression was found in vehicle or low-dose groups. No overt toxicity effect was observed at the four doses, and YM101 treatment had no significant impact on body weight (Fig. 6b). Hereto, we used 9 mg/kg as the optimal dose of YM101 in the following in vivo studies. Correspondingly, equivalent mole anti-PD-L1 (6.6 mg/kg) and anti-TGF-β (6.6 mg/kg) were used as controls.

Then, we compared the anti-tumor effect of YM101 with other controls, including vehicle, anti-TGF-β, and anti-PD-L1. In the EMT-6 orthotopic tumor model, anti-TGF-β didn’t exhibit a significant anti-tumor effect while anti-PD-L1 treatment partially suppressed tumor growth. The anti-tumor activity of YM101 was superior to vehicle (*p* < 0.0001), anti-TGF-β (*p* < 0.0001), anti-PD-L1 (*p* < 0.05) (Fig. 6c, d). The tumor weight in YM101 treatment group was significantly lower compared with vehicle (*p* < 0.001), anti-TGF-β (*p* < 0.001), and anti-PD-L1 (*p* < 0.01) groups (Fig. 6e).

Besides, we evaluated the anti-tumor activity of YM101 in the CT26 tumor model. In this model, although anti-PD-L1 effectively inhibited the tumor growth, the tumor volume was lowest in the YM101-treated group, relative to vehicle (*p* < 0.0001), anti-TGF-β (*p* < 0.0001), and anti-PD-L1 (*p* < 0.05) (Fig. 6f, g). The tumor weight in YM101-treated group was lower than vehicle (*p* < 0.01), anti-TGF-β (*p* < 0.001), and anti-PD-L1 (*p* = 0.09) with or on the verge of statistical significance (Fig. 6h).

Moreover, we compared the efficacy of YM101 with that of anti-PD-L1 plus anti-TGF-β treatment in the 3LL model. The anti-tumor effect of YM101 was slightly superior to that of the combination therapy (*p* = 0.29) (Fig. 6i, j). In addition, the ratio of complete regression was higher in YM101 (3 of 8) than that in the vehicle (0 of 8), anti-PD-L1 (0 of 8), anti-PD-L1 (0 of 8), and the combination treatment (1 of 8) group (Fig. 6i). The tumor weight in YM101-treated group was lowest, relatively to the other groups (Fig. 6k). In the repeated 3LL challenge experiment, after receiving 6 doses of treatments, mice were followed up for 14 days. Compared to anti-TGF-β and anti-PD-L1 treatment, YM101 lengthened mice survival with or on the verge of statistical significance (*p* < 0.001 and *p* = 0.06, respectively) (Additional file 1: S3a, b). In the 3LL rechallenge assay, YM101 exhibited a durable anti-tumor activity and markedly retarded tumor growth (Fig. 6l–o).

### YM101 promoted T cell infiltration and reshaped the TME

**Fig. 7:**
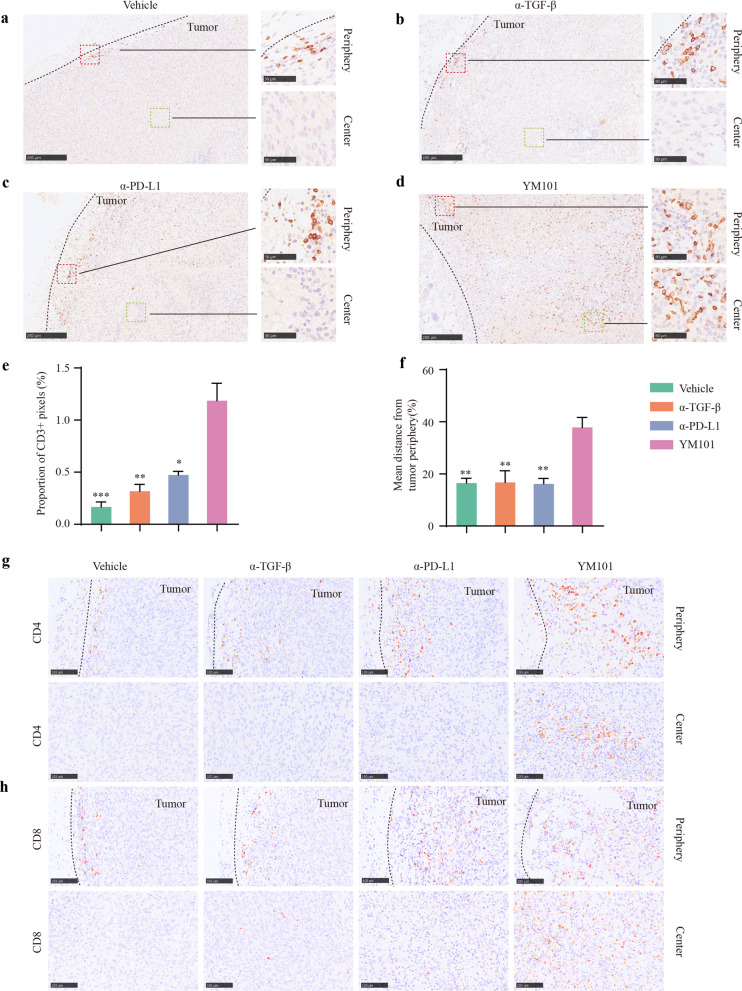
Immunohistochemical staining assays to measure the infiltration of T cells in EMT-6 tumors. **a**–**d** The presentative images of tumor-infiltrating CD3^+^ cells in the tumor periphery and the tumor center. Scale bars, 250 μm or 50 μm. **e** The quantitative analyses for the number of tumor-infiltrating CD3^+^ cells, and the proportion of CD3^+^ area was used. **f** The quantitative analysis for the infiltration depth. The infiltration depth of CD3^+^ cells was calculated by the mean nearest distance of all CD3^+^ cells to the tumor border. The mean nearest distance was scaled by the distance between the corresponding tumor border to tumor center. **g**, **h** The presentative images of tumor-infiltrating CD4^+^ and CD8^+^ cells in the tumor periphery and the tumor center. Scale bars, 100 μm. *p < 0.05, **p < 0.01, ***p < 0.001, and ****p < 0.0001 denote the significant difference relative to YM101 treatment. α-TGF-β: anti-TGF-β, α-PD-L1: anti-PD-L1

TGF-β signaling undermines the penetration of T cells into the tumor center [30]. To explore the effect of YM101 on T cell infiltration, we performed anti-CD3, anti-CD4, anti-CD8 IHC staining assays using EMT-6 tumor samples. Relative to the vehicle, anti-TGF-β, and anti-PD-L1 monotherapy, YM101 treatment remarkably facilitated T cells to infiltrate into the tumor center (Fig. 7a–d). Further quantitative analysis indicated that YM101 not only increased the number of tumor-infiltrating T cells (Fig. 7e) but also altered the localization of T cells (Fig. 7f). In contrast, we found the single antibody treatment had no significant effect on T cell penetration. Anti-CD4 and anti-CD8 IHC stainings showed that although YM101 had a modest effect on the number of CD4^+^ and CD8^+^ T cells in the peritumoral stroma (Fig. 7g, h), YM101 markedly increased the quantity of T cells in tumor center (Fig. 7g, h). Our results demonstrated that YM101 could promote T cell infiltration and provide an optimal T cell positioning to enhance the anti-tumor immune response.

**Fig. 8:**
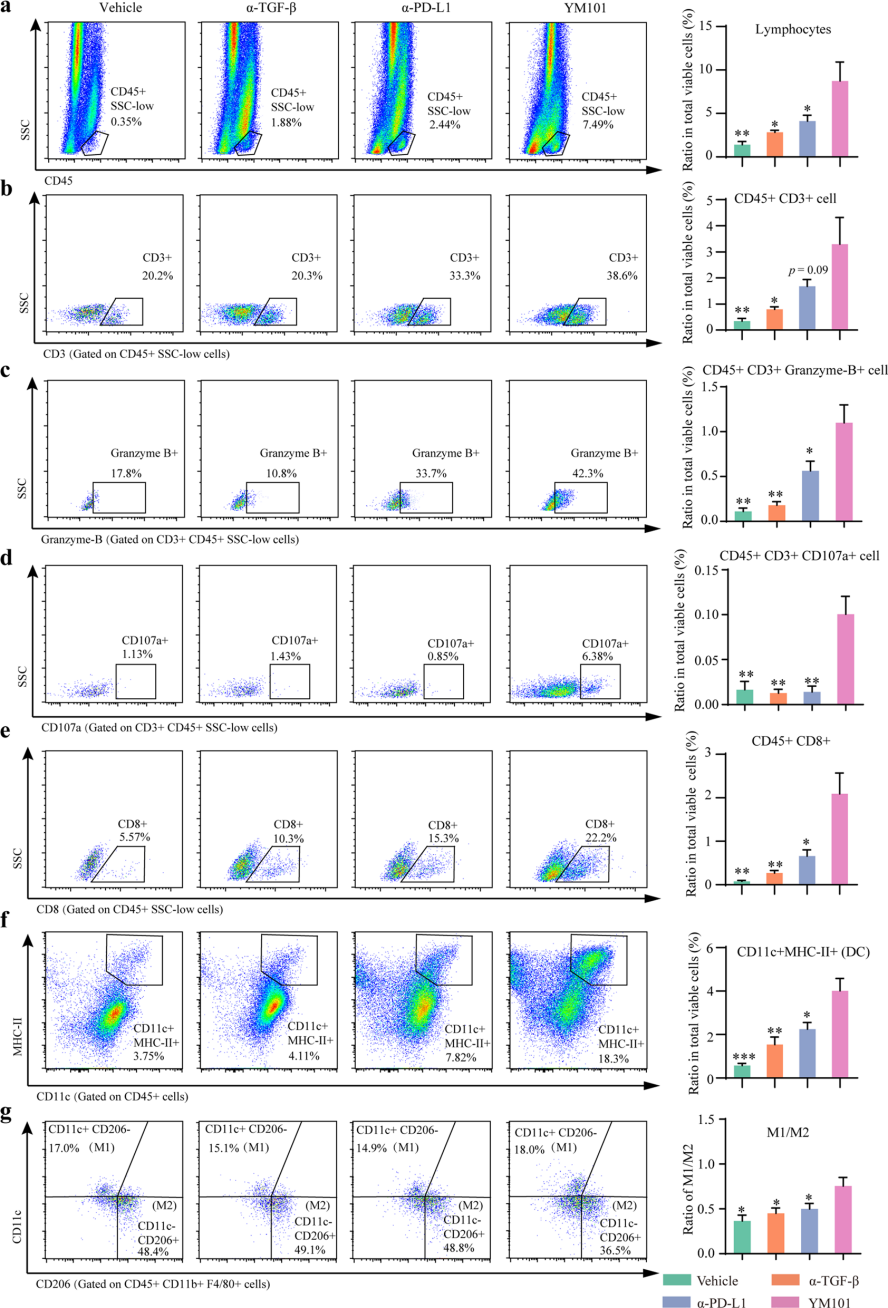
Flow cytometry assays to analyze the tumor immune microenvironment in EMT-6 tumors. Representative images of **a** tumor-infiltrating lymphocytes, **b** T cells, **c** granzyme B^+^ T cells, **d** CD107a^+^ T cells, **e** CD8^+^ T cells, **f** dendritic cells (DCs), **g** macrophages. The relative quantitative analysis was performed by the ratio of tumor-infiltrating immune cells to total alive cells in the prepared cell suspension. *p < 0.05, **p < 0.01, ***p < 0.001, and ****p < 0.0001 denote the significant difference relative to YM101 treatment. α-TGF-β: anti-TGF-β, α-PD-L1: anti-PD-L1

In the EMT-6 bearing mouse model, YM101 increased the density (the ratio in all viable cells) of tumor infiltrating lymphocytes (TILs) compared to vehicle (*p* < 0.01), anti-TGF-β (*p* < 0.05), and anti-PD-L1 (*p* < 0.05) (Fig. 8a). In addition, YM101 increased the density of T cells compared to vehicle (*p* < 0.01), anti-TGF-β (*p* < 0.05), and anti-PD-L1 (*p* = 0.09) with or on the verge of statistical significance (Fig. 8b). Apart from the number of T cells, we found YM101 simultaneously enhanced the cytotoxic activity of T cells. The densities of granzyme B^+^ and CD107a^+^ T cells were elevated after YM101 treatment, relative to vehicle (*p* < 0.01, *p* < 0.01, respectively), anti-TGF-β (*p* < 0.01, *p* < 0.01, respectively), anti-PD-L1 (*p* < 0.05, *p* < 0.01, respectively) (Fig. 8c, d). Moreover, YM101 increased the density of tumor infiltrating CD8^+^ T cells, relative to vehicle (*p* < 0.01), anti-TGF-β (*p* < 0.01), anti-PD-L1 (*p* < 0.05) (Fig. 8e).

In addition to T cells, YM101 treatment increased the density of DCs, which were the main professional antigen-presenting cells in the TME. The density of DCs in YM101-treated tumors was higher than that in vehicle-treated (*p* < 0.001), anti-TGF-β-treated (*p* < 0.01), and anti-PD-L1-treated tumors (*p* < 0.05) (Fig. 8f). Furthermore, YM101 regulated the polarization of macrophages and increased the ratio of M1-like macrophage (M1) to M2-like macrophage (M2). YM101 increased the ratio of M1/M2, relative to vehicle (*p* < 0.05), anti-TGF-β (*p* < 0.05), and anti-PD-L1 (*p* < 0.05) (Fig. 8g).

### YM101 altered the expression profile of immune-related genes

**Fig. 9:**
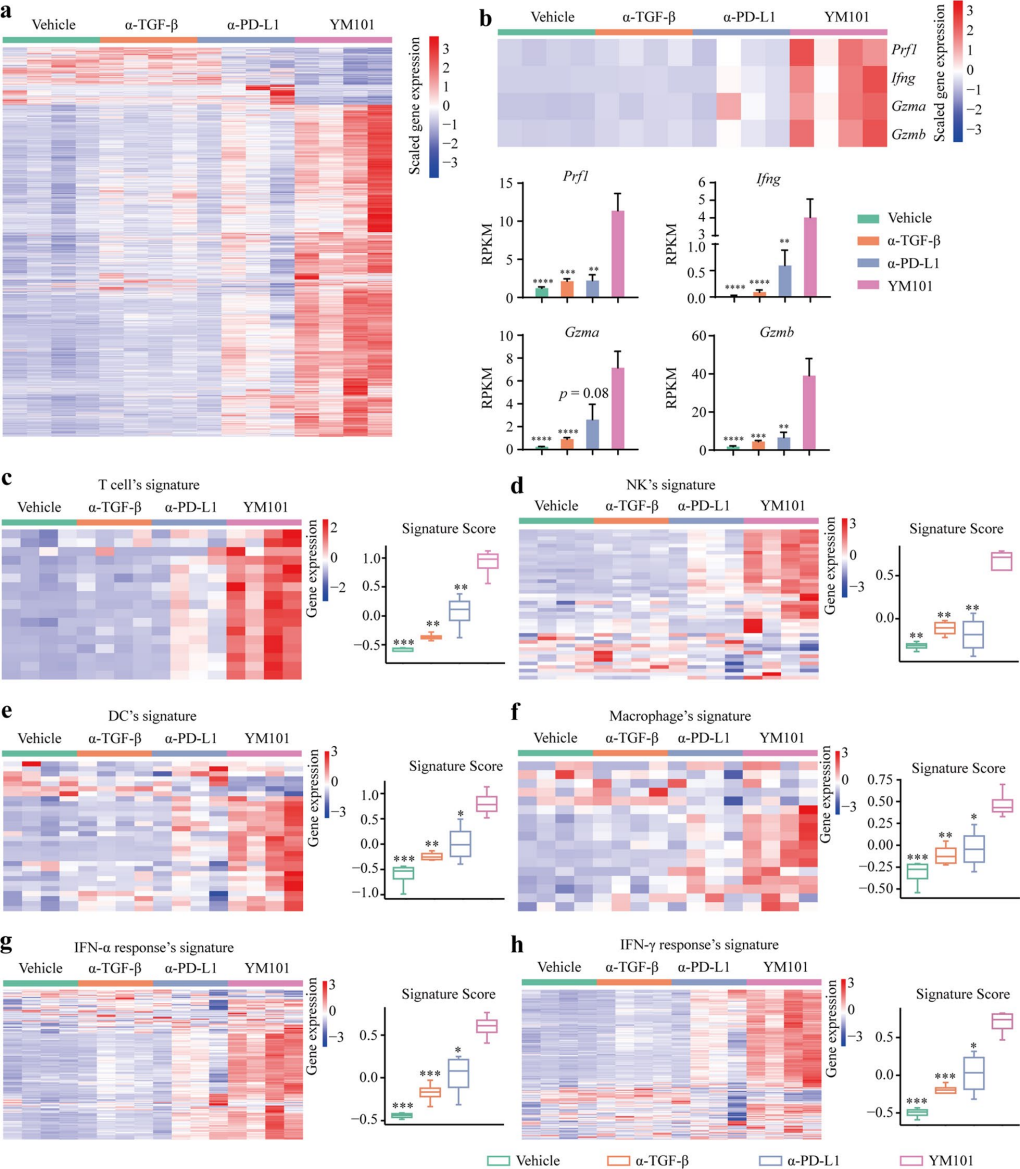
RNA-seq to explore the immune landscape of EMT-6 tumors. **a** The heat map of the expression levels of all differentially expressed genes (fold change > 2, *p* < 0.05). **b** The expression levels [The Reads Per Kilobase per Million mapped reads (RPKM)] of *Prf1*, *Ifng*, *Gzma*, and *Gzmb*. **c**–**h** The expression levels of genes in T cell’s signature, NK’s signature, dendritic cell (DC)’s signature, macrophage’s signature, IFN-α response’s signature, IFN-γ response’s signature. *p < 0.05, **p < 0.01, ***p < 0.001, and ****p < 0.0001 denote the significant difference relative to YM101 treatment. α-TGF-β: anti-TGF-β, α-PD-L1: anti-PD-L1

To explore the effect of YM101 on gene expression, we conducted RNA-seq assays using EMT-6 tumors. DEG analysis revealed that 2651, 1865, and 1173 genes were differently expressed in vehicle, anti-TGF-β, and anti-PD-L1 groups, relative to YM101 group (Fig. 9a). Our results indicated that YM101′s anti-PD-L1 moiety caused most gene expression changes. Among all DEGs, some cytotoxicity-related genes such as *Prf1* (perforin), *Ifng* (interferon), *Gzma* (granzyme A), and *Gzmb* (granzyme B) were upregulated in YM101-treated group (Fig. 9b).

To evaluate the effect of YM101 on the components of the TME, we calculated the scores of multiple immune signatures. The scores of the signatures of T cell, NK, DC, macrophage, IFN-α response, and IFN-γ response were markedly increased in the YM101 group (Fig. 9c–h).

### YM101 inhibited TGF-β-Smad signaling, reduced collagen expression and reversed EMT in mouse model

**Fig. 10:**
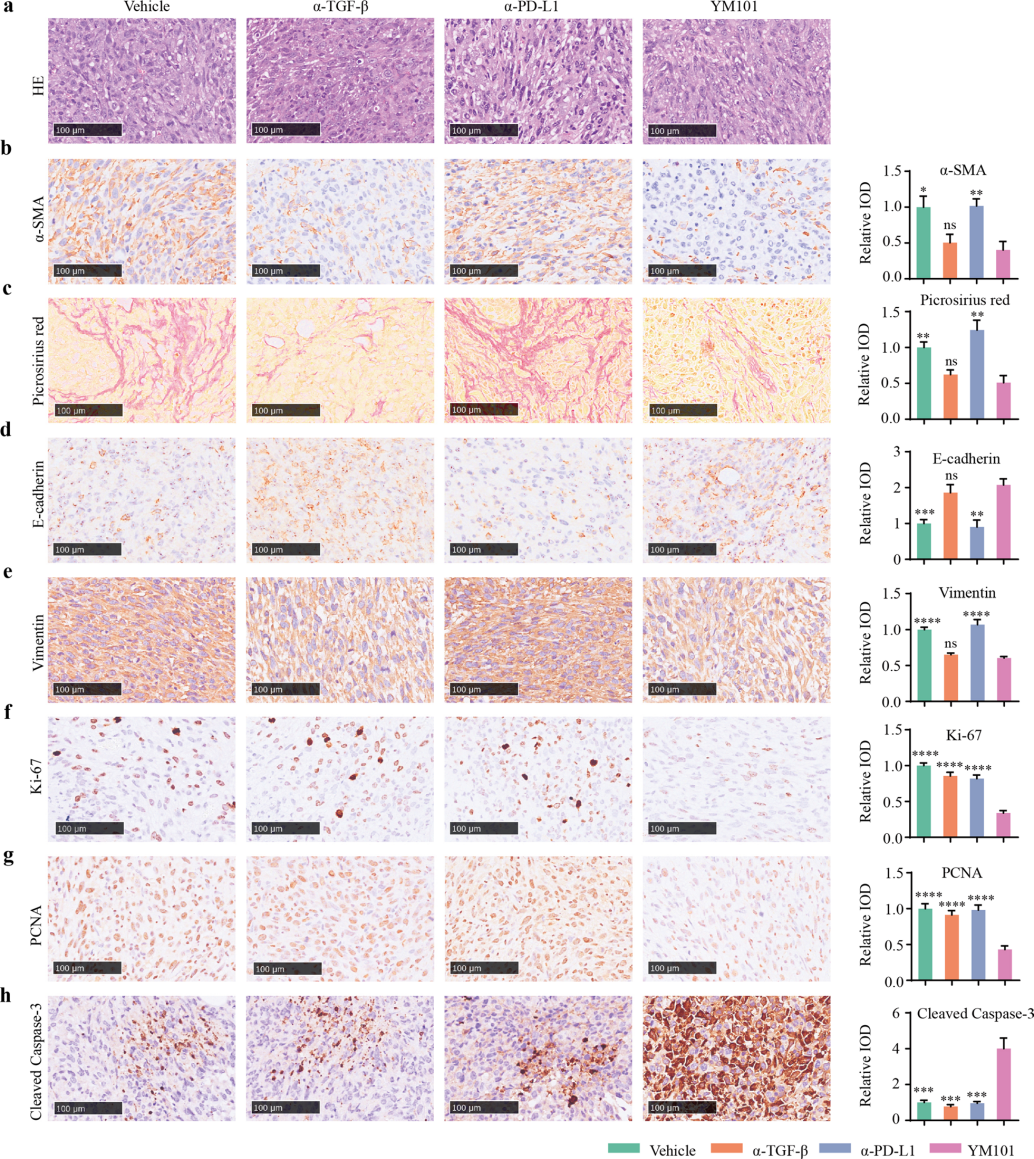
Immunohistochemical staining to evaluate the activity of carcinoma-associated fibroblast, the status of mediated epithelial-mesenchymal transition of cancer cells, as well as the proliferation and apoptosis of cancer cells. **a** H&E staining. **b** Anti-α-SMA staining. **c** Picrosirius red staining. **d** Anti-E-cadherin staining. **e** Anti-Vimentin staining. **f** Anti-Ki-67 staining. **g** Anti-PCNA staining. **h** Anti-cleaved-Caspase 3. For quantitative analysis, the integral optical density (IOD) of values the IHC stainings were calculated. *p < 0.05, **p < 0.01, ***p < 0.001, and ****p < 0.0001 denote the significant difference relative to YM101 treatment. Scale bars, 100 μm. α-TGF-β: anti-TGF-β, α-PD-L1: anti-PD-L1

The results of anti-TGF-β1 and anti-p-Smad3 IHC assays showed that, relative to vehicle and anti-PD-L1, YM101 lowered the expressions of TGF-β1 and p-Smad3 in the EMT-6 tumor model (Additional file 1: Figure S4a, b). α-SMA is a classic marker of CAF [60]. In the EMT-6 tumor, as a contrast to vehicle and anti-PD-L1, YM101 significantly reduced α-SMA expression (Fig. 10a, b). Then, we performed a picrosirius red staining to measure the collagen deposition. Compared with vehicle- and anti-PD-L1-treated tumors, the collagen deposition was markedly decreased in YM101-treated tumors (Fig. 10c). Our results indicated that YM101 retrained the CAF activity and reduced the collagen production by anti-TGF-β moiety.

Moreover, we investigated the effect of YM101 treatment on EMT in the EMT-6 tumor model. We utilized an epithelial marker (E-cadherin) and a mesenchymal marker (Vimentin) to assess EMT phenotype. The results of IHC staining showed that YM101 upregulated E-cadherin but downregulated Vimentin (Fig. 10d, e). A similar transformation was observed in anti-TGF-β-treated tumors.

### YM101 inhibited tumor cell’s proliferation and promoted tumor cells’ apoptosis

To investigate the effect of YM101-stimulated immunity on tumor cells, we conducted IHC staining for anti-Ki67, anti-PCNA, anti-cleaved-Caspase 3 using EMT-6 tumor samples. We found that YM101 decreased the expressions of Ki67 and PCNA but increased the expression of cleaved-Caspase 3 in tumors (Fig. 10f–h). The results indicated YM101 suppressed the tumor cells’ proliferation and promoted tumor cells’ apoptosis, which might relate to the enhanced cytotoxicity of anti-tumor immunity.

## Discussion

For advanced cancers, TGF-β transforms from a tumor-suppressive cytokine to a tumor-promoting cytokine. Under the selective pressure, some cancer clones acquire loss-of-function mutations in TGF-β pathway. Alternatively, the downstream pathways of TGF-β signal are rewired and decoupled from apoptosis in cancer cells [32]. In this context, a TGF-β-enriched TME fosters the non-lethal EMT and promotes cancer metastasis. Besides acting on cancer cells, TGF-β could directly impair the functions of immune cells and facilitate immune evasion. Hereto, targeting TGF-β signal might be favorable to control tumor growth.

As far as we knew, PD-L1 expression are regulated at the levels of transcription, post-transcription, post-translation [61]. The existing anti-tumor immune response could be impaired by several factors in the TME, including but not limited to the PD-1/PD-L1 axis and TGF-β signaling [20]. In the Cancer-Immunity Cycle model, the PD-1/PD-L1 and TGF-β modulate several steps of anti-tumor immunity such as infiltration of T cells and killing of cancer cells. Because Cancer-Immunity Cycle contains a series of stepwise events, each step in the cycle could determine the eventual magnitude of the anti-tumor immune response. Based on the synergistic effect of TGF-β and PD-1/PD-L1 pathways in cancer immune escape, we developed YM101, which could simultaneously target these two pathways.

The binding affinities of YM101 to TGF-β and PD-L1 were close to or slightly weaker than the parent monoclonal antibodies. In the present study, YM101 bound specifically to three TGF-β isoforms and PD-L1. In parallel, YM101 effectively reversed the biological effects of TGF-β and PD-1/PD-L1. The results of in vitro studies demonstrated that YM101 counteracted TGF-β-mediated Treg differentiation, proliferation inhibition in T cells, and EMT in cancer cells. During T cell activation, PD-1/PD-L1-mediated inhibitory effects on T cells could be overturned by YM101.

**Fig. 11:**
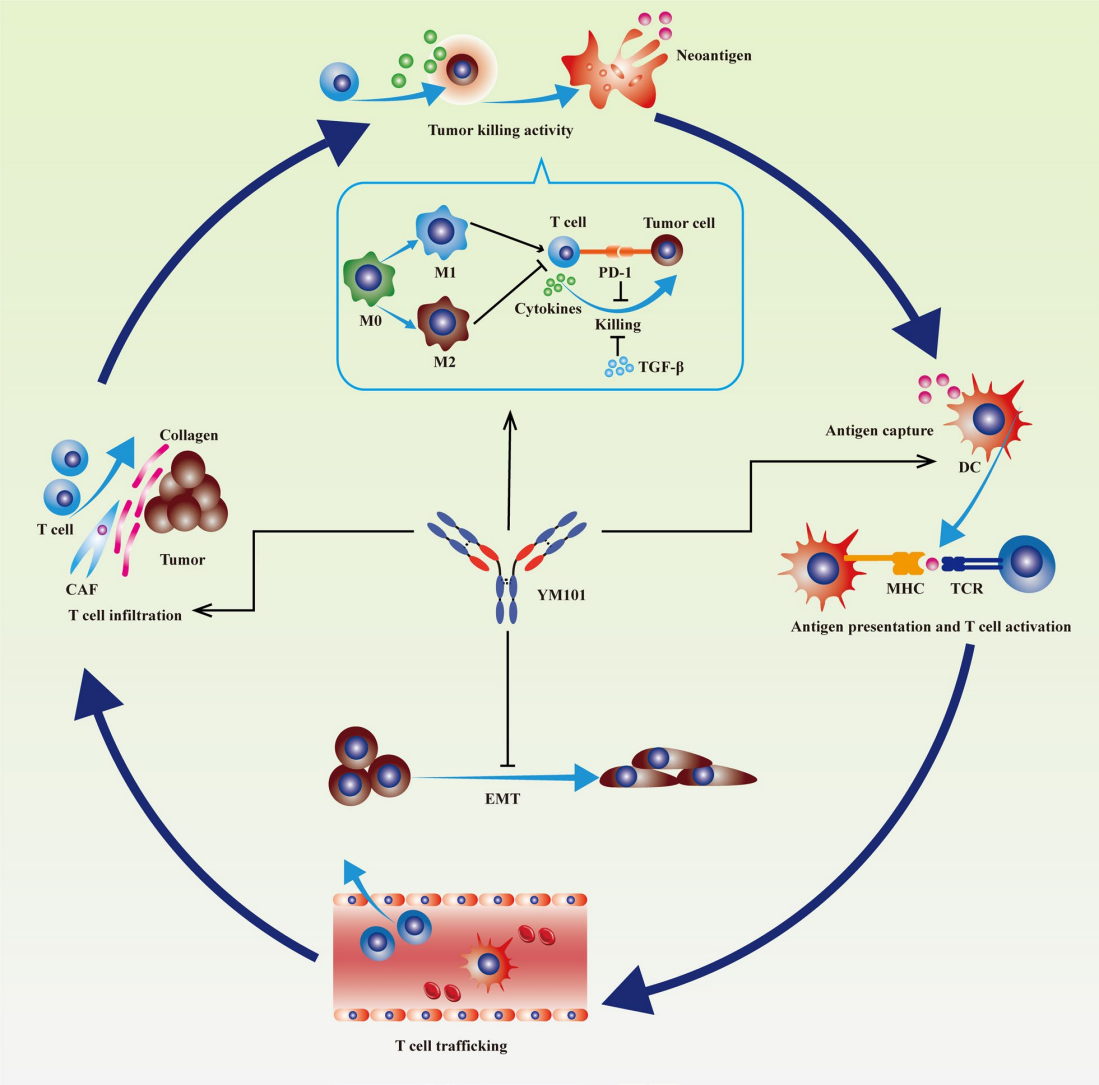
Schematic diagram showing the effect of YM101 on Cancer-Immunity Cycle and tumor cells. Firstly, YM101 promoted T cell infiltration by restraining the activity of carcinoma-associated fibroblast (CAF). Secondly, YM101 enhanced the tumor-killing activity of T cells by blocking PD-1/PD-L1 and naturalizing TGF-β. Thirdly, YM101 altered the polarization of macrophages and increased the ratio of M1/M2. Besides, YM101 increased the density of dendritic cells (DCs) which would be favorable to antigen presentation in the TME. Lastly, YM101 counteracted epithelial-mesenchymal transition (EMT) in tumor cells

In multiple murine models, the anti-tumor effect of YM101 was superior to the single anti-TGF-β or anti-PD-L1 treatment. Given that the anti-tumor activity of YM101 might depend on immunity, we investigated the influence of YM101 on the TME using the EMT-6 tumor model. The results of IHC, flow cytometry, and RNA-seq assays indicated that YM101 substantially increased the number of TILs and DCs, elevated the ratio of M1/M2, as well as promoted cytokine production in T cells, relative to control groups. Generally, YM101 normalized the immune-deficient TME and exhibited a robust anti-tumor activity (Fig. 11).

In some models such as EMT-6 tumors, the efficacy of individual anti-PD-L1 treatment was moderate. The previous studies found that in this high TGF-β tumor model, TGF-β undermined anti-tumor immunity by promoting the exclusion of T cells [30]. Activated TGF-β signaling in CAFs increased collagen generation and hampered T cell infiltration [30]. We found in the EMT-6 model, YM101 significantly decreased collagen deposition and increased T cell infiltration into the tumor center (termed immune-inflamed phenotype). On the contrary, in anti-PD-L1 treated tumors, T cells were mainly located in tumor peripheral but rarely infiltrated into the tumor center (termed immune-excluded phenotype). The transformation from immune-excluded to immune-inflamed phenotype might contribute to the advantages of YM101 in treatment effect.

The immune normalization strategy aims to recover the blocked anti-tumor immune response. In partial patients, normalizing a single vital pathway such as PD-1/PD-L1 is sufficient to trigger to reshape the TME [62]. However, for most patients, immune deficiency or dysregulation in the TME is often multifaceted, and correcting other defects might be necessary to overcome the resistance to anti-PD-1/PD-L1 therapy. Based on the fact that TGF-β is the dominant inhibitory pathway, the dual blockade of TGF-β and PD-1/PD-L1 by YM101 could effectively alter the ‘cancer-immunity set point,’ converting immune tolerance to activated T cell-immunity. From this perspective, YM101 would be an important complement to the current immunotherapy strategies.

Prior to YM101, a bifunctional fusion antibody targeting TGF-β and PD-L1 (M7824) had been developed. The results of preclinical studies of M7824 showed that the dual blockade of TGF-β and PD-L1 was feasible in cancer treatment [54]. Moreover, the results of the phase I studies of M7824 indicated that this dual blockade therapeutic strategy was successful in clinical practice, especially for PD-L1-high NSCLC patients (objective response rate: 85.7%) [63, 64]. In this study, YM101 is a novel antibody developed with the bispecific antibody development platform Check-BODY™. Different from the bi-functional fusion antibody, we try to simultaneously block these two signaling pathways by a bispecific antibody, which is an innovation from the perspective of production technology. The construction of YM101 is a pilot experiment, which provides a rationale to develop the anti-TGF-β/human PD-L1 bispecific antibody. Besides, based on Check-BODY™ platform, we believe that more bispecific antibodies could be developed to simultaneously block two vital signal pathways in cancer, which would have a strategic advantage over the combination therapy of two single antibodies.

## Conclusion

In conclusion, we developed a novel bispecific antibody YM101, which simultaneously blocked TGF-β and PD-1/PD-L1 pathways. YM101 exhibited a potent anti-tumor activity, even in the murine models in which single anti-TGF-β or anti-PD-L1 treatment did not trigger substantial tumor regression. Further investigations on the TME revealed that YM101 promoted the formation of immune inflamed tumor, normalized the dysregulated anti-tumor immunity, and provided an immunosupportive TME. Based on the encouraging results of this pilot experiment, it is promising to further develop the anti-TGF-β/human PD-L1 bispecific antibody. As increased TGF-β is a dominantly immune inhibitory pathway in multiple types of cancer, an anti-TGF-β/PD-L1 bispecific antibody might provide a choice for cancer patients resistant to immune checkpoint inhibitors.

## Supplementary Information

**Additional file 1.** Supplementary tables and figures.

## Data Availability

The dataset generated during the current study is available from the corresponding author on reasonable request.

## Abbreviations

PD-1: Programmed cell death protein 1
TME: Tumor microenvironment
TGF-β: Transforming growth factor-beta
Treg: Regulatory T
DC: Dendritic cells
FBS: Fetal bovine serum
SDS-PAGE: Sodium dodecyl sulfate–polyacrylamide gel electrophoresis
CE-SDS: Capillary electrophoresis with sodium dodecylsulfate
EC_50_: Half maximal effective concentration
IC_50_: Half maximal inhibitory concentration
ELISA: Enzyme-linked immunosorbent assay
IFN-γ: Interferon-γ
EMT: Epithelial-mesenchymal transition
ROI: Regions of interest
IOD: Integral optical density
DEG: Differently expressed gene
CAF: Carcinoma-associated fibroblast
M1: M1-like macrophage
M2: M2-like macrophage
