# Supplementary material for: The construction, expression, and enhanced anti-tumor activity of YM101: a bispecific antibody simultaneously targeting TGF-β and PD-L1
Source: J Hematol Oncol. 2021 Feb 16;14:27. doi: 10.1186/s13045-021-01045-x (PMC7885589; doi:10.1186/s13045-021-01045-x)
Supplement: Supplementary file 1 — Additional file 1. Supplementary tables and figures. [file 13045_2021_1045_MOESM1_ESM.pdf]

The construction, expression, and enhanced anti-tumor activity of YM101: a bispecific antibody simultaneously targeting TGF-β and PD-L1

Ming Yi[1], Jing Zhang[2], Anping Li[3], Mengke Niu[1,3], Yongxiang Yan[2], Ying Jiao[1], Suxia Luo[3], Pengfei Zhou[2*], Kongming Wu[1,3*]

1. Department of Oncology, Tongji Hospital of Tongji Medical College, Huazhong University of Science and Technology, 1095 Jiefang Avenue, Wuhan, 430030, People's Republic of China.
2. Wuhan YZY Biopharma Co., Ltd, Biolake, C2-1, No.666 Gaoxin Road, Wuhan, 430075, People's Republic of China.
3. Department of Medical Oncology, The Affiliated Cancer Hospital of Zhengzhou University & Henan Cancer Hospital, Zhengzhou, 450008, People's Republic of China.

*** Co-corresponding authors**

Kongming Wu, Department of Oncology, Tongji Hospital of Tongji Medical College, Huazhong University of Science and Technology, 1095 Jiefang Avenue, Wuhan, 430030, People's Republic of China. E-mail: wukm_lab@163.com.

Pengfei Zhou, Wuhan YZY Biopharma Co., Ltd, Biolake, C2-1, No.666 Gaoxin Road, Wuhan, 430075, People's Republic of China. E-mail: pfzhou@yzybio.com.

Author E-mails:

Ming Yi: d201981836@hust.edu.cn;

Jing Zhang: zhangjing@yzybio.com;

Anping Li: li_anping@yahoo.com;

Mengke Niu: niumengke9505@163.com;

Yongxiang Yan: yanyongxiang@yzybio.com;

Ying Jiao: 754493026@qq.com;

Suxia Luo: luosxrm@163.com;

Pengfei Zhou: pfzhou@yzybio.com;

Kongming Wu: wukm_lab@163.com.

Figure S1: The molecular weight of YM101. (a) The molecular weight of the intact antibody. **(b-c)** The molecular weights of the light chain and heavy chain.

Figure S2: Western blotting assays to measure the effect of YM101 on Smad-independent MAPK pathway. After treatment with TGF-β1 (10 ng/ml) plus antibodies, phosphorylated Erk1/2 were detected.

Figure S3: The effect of YM101 treatment on survival in the 3LL tumor model. 3LL-bearing mice received 6 doses of YM101 treatment and then were followed up for 2 weeks. **(a)** The survival curves of 3LL-bearing mice. **(b)** The ratioes of complete regression in different treatment groups.

Figure S4: Immunohistochemical staining to evaluate the activity of TGF-β signaling pathway in the EMT-6 tumor model. (a) Anti-TGF-β1 IHC staining. **(b)** Anti-p-Smad3 IHC staining.

Figure S1

a

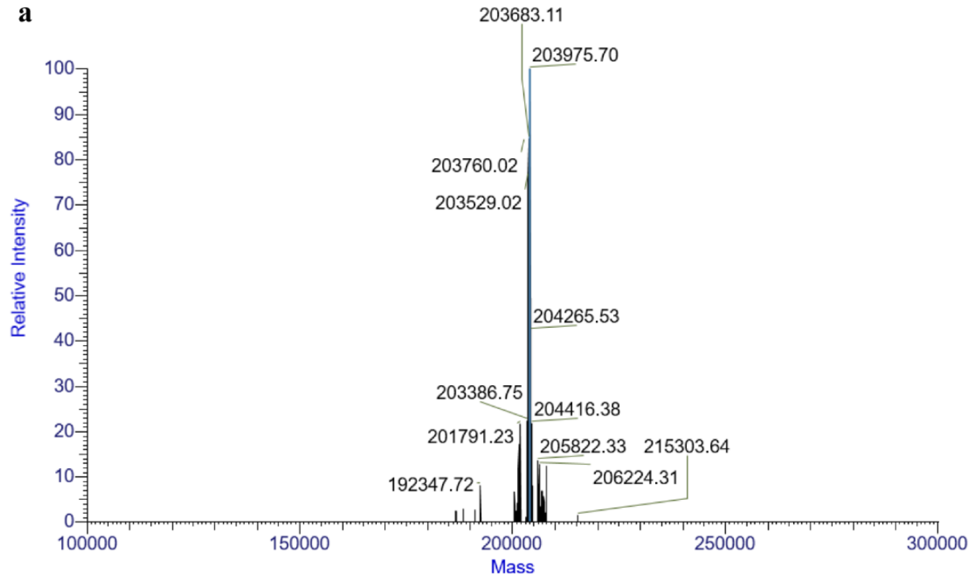

b

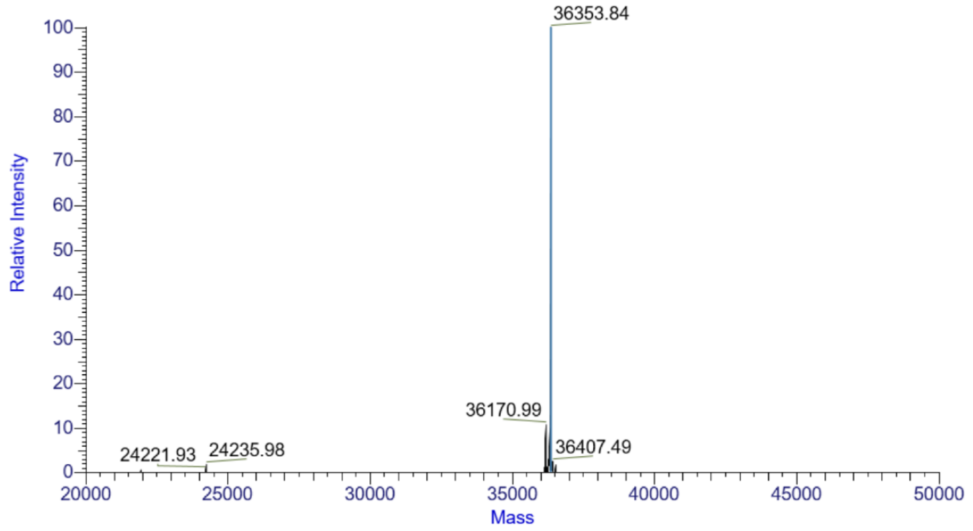

c

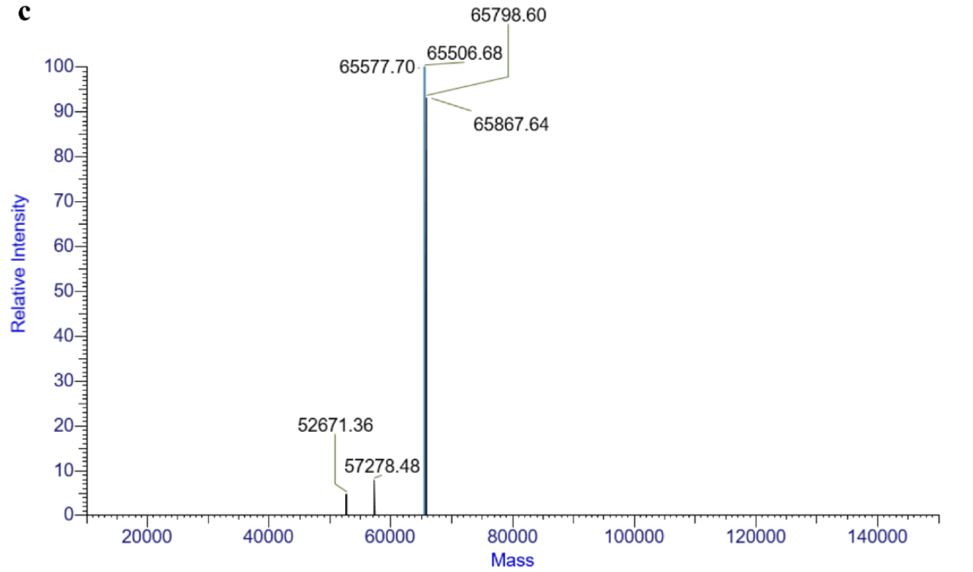

Fig S2

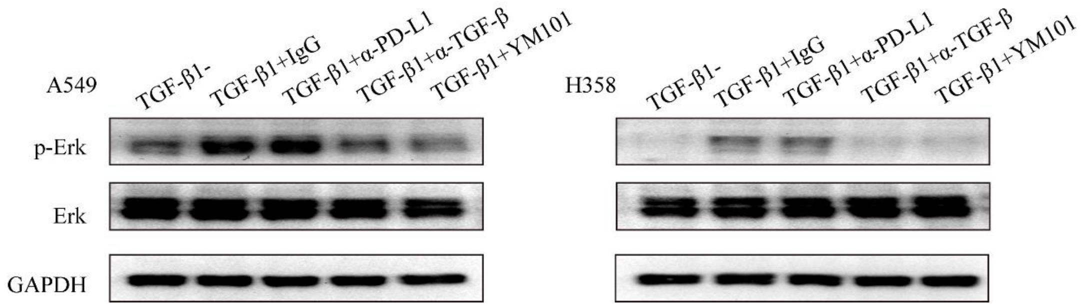

Fig S3

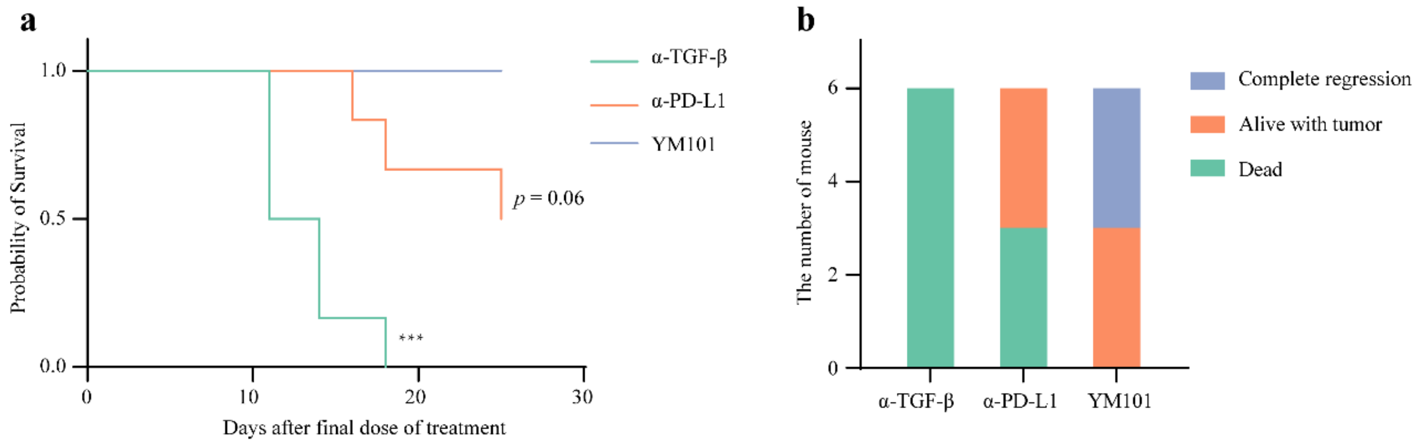

Fig S4

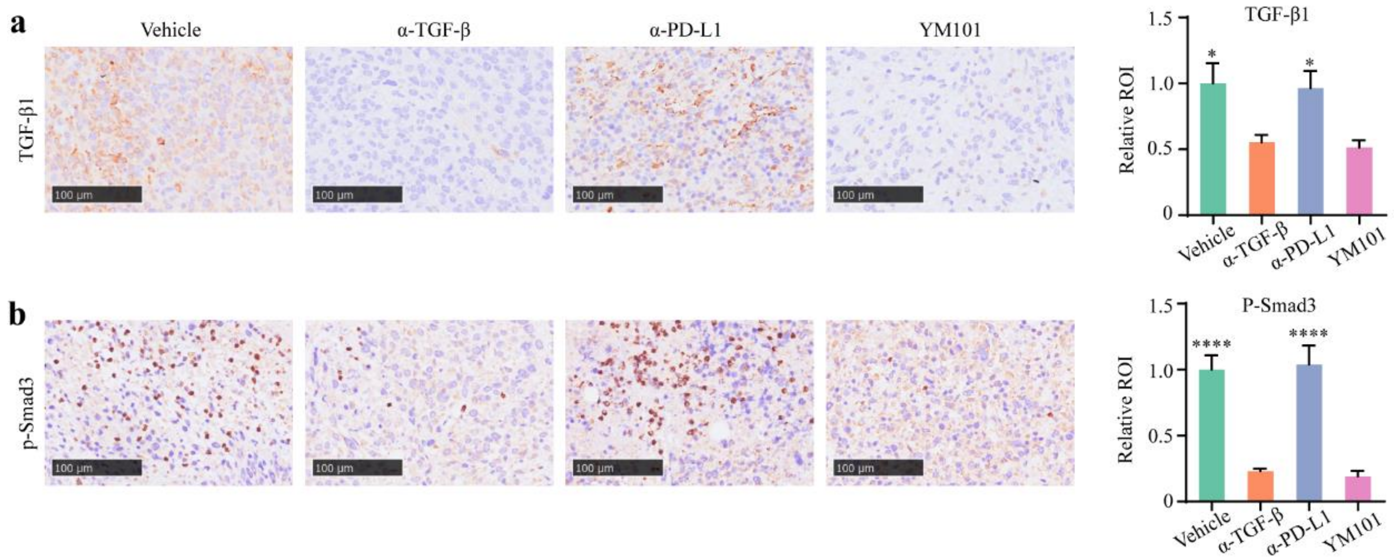

Table S1: Gene lists of immune signatures in transcriptome analysis.

The immune signatures were designed according to a previous study [1].

Immune signatures	Genes
T cell	*Bcl11b, Cd2, Cd28, Cd3d, Cd3e, Cd3g, Cd6, Cd96, Gimap3, Gimap5, Itm2a, Lck, Ncald, Prkcq, Sh2d1a, Skap1, Trat1*
NK cell	*Bzrap1, Cacna2d2, Cd247, Cd7, Colq, Ctsw, Dll1, Eomes, Fam179a, Fasl, Fez1, Gk5, Gpr56, Gtf3c1, Gzmd, Gzme, Gzmf, Gzmg, Gzmm, Il12rb2, Il2rb, Kir3dl1, Kir3dl2, Klrc1, Klrc2, Klrc3, Klrd1, Lair1, Myom2, Ncald, Nkg7, Phldb2, Plekhf1, Prf1, Samd3, Stat4, Tbc1d31, Tbx21, Tigit, Tox, Tsen54, Txk, Yes1*
Dendritic cell	*C1qc, Ccdc88a, Ccl2, Ccl3, Cd300e, Cd86, Clec1a, Clec4b1, Clec5a, Csf2ra, Fam49a, Fcgr1, Fn1, Fstl1, Gpx3, H2-Aa, Inhba, Lgmn, Ngfr, Pdgfrl, Pdpn, Prkar2b, Siglec1, Slamf9, Stab1, Thbd, Tnfaip2, Ttyh2, Ubd, Vcam1*
Macrophage	*Aif1, Ccl1, Ccl26, Cd163, Cd300lb, Cnr1, Cnr2, Cpm, Csf3r, Eng, Igf1, Il34, L1cam, Lilra5, Lrp1, Ms4a7, Trem1, Treml1*
IFN-α response	*1700057G04Rik, 2200002J24Rik, AC132444.1, AC168977.1, Adar, B2m, Batf2, Bst2, C1s, Casp1, Casp8, Ccrl2, Cd47, Cd74, Cmpk2, Cmtr1, Cnp, Csf1, Cxcl10, Cxcl11, Cxcl15, Ddx60, Dhx58, Eif2ak2, Elf1, Epsti1, Fam46a, Fibin, Gbp11, Gbp2, Gbp3, Gbp4, Gbp5, Gbp7, Gbp8, Gbp9, Gm11127, Gm7030, Gm8909, Gmpr, Gmpr2, H2-D1, H2-K1, H2-M1, H2-M10.1, H2-M10.2, H2-M10.3, H2-M10.4, H2-M10.5, H2-M10.6, H2-M11, H2-M2, H2-M3, H2-M5, H2-M9, H2-Q1, H2-Q10, H2-Q2, H2-Q4, H2-Q6, H2-Q7, H2-T10, H2-T22, H2-T23, H2-T24, H2-T3, Helz2, Herc3, Herc6, I830012O16Rik, Ifi27, Ifi27l2a, Ifi27l2b, Ifi35, Ifi44, Ifih1, Ifit2, Ifit3, Ifitm1, Ifitm2, Ifitm3, Ifitm5, Ifitm6, Ifitm7, Il15, Il4ra, Il7, Irf1, Irf2, Irf7, Irf9, Isg15, Isg20, Lamp3, Lap3, Lgals3bp, Lpar6, Ly6e, Mov10, Mvb12a, Mx2, Ncoa7, Nmi, Nub1, Oas1a, Oas1b, Oas1c, Oas1d, Oas1e, Oas1f, Oas1g, Oas1h, Oas2, Oas3, Oasl1, Oasl2, Ogfr, Parp12, Parp14, Parp9, Plscr1, Plscr2, Plscr5, Pnpt1, Procr, Psma3, Psmb11, Psmb5, Psmb8, Psmb9, Psme1, Psme2, Psme2b, Ripk2, Rnf31, Rsad2, Rtp4, Samd9l, Sell, Slc25a28, Sp110, Stat2, Tap1, Tdrd7, Tmem140, Trafd1, Trim12a, Trim12c, Trim14, Trim21, Trim25, Trim26, Trim30a, Trim30b, Trim30d, Trim31, Trim34a, Trim34b, Trim43a, Trim43b, Trim43c, Trim5, Trim6, Txnip, Uba1, Uba1y, Uba7, Ube2l6, Usp18, Wars*
IFN-γ response	*1700057G04Rik, 2010002M12Rik, 2200002J24Rik, 4922502D21Rik, 4930525M21Rik, AC132444.1, AC168977.1, Adar, Apol6, Apol9a, Arid5b, Arl4a, Arl4c, Arl4d, B2m, Bank1, Batf2, Bpgm, Bst2, Btg1, C1ra, C1rb, C1s, Casp1, Casp12, Casp3, Casp4, Casp7, Casp8, Ccl11, Ccl12, Ccl2, Ccl5, Ccl7, Ccl8, Cd274, Cd38, Cd40, Cd69, Cd74, Cd86, Cdkn1a, Cfb, Cfh, Cfhr1, Cfhr2, Ciita, Cmklr1, Cmpk2, Cmtr1, Csf2rb, Csf2rb2, Cxcl10, Cxcl11, Cxcl15, Cxcl9, Ddx58, Ddx60, Dhx58, Eif2ak2, Eif4e3,*

	Epas1, Epsti1, Fas, Fbxo39, Fcgr1, Fgl2, Fibin, Fpr-rs3, Fpr-rs4, Fpr-rs6, Fpr1, Fpr2, Fpr3, Gbp11, Gbp2, Gbp3, Gbp4, Gbp5, Gbp7, Gbp8, Gbp9, Gch1, Gm11127, Gm14446, Gm4788, Gm7030, Gm8909, Gpr18, Gzma, H2-Aa, H2-Ab1, H2-D1, H2-DMa, H2-Eb1, H2-Eb2, H2-K1, H2-M1, H2-M10.1, H2-M10.2, H2-M10.3, H2-M10.4, H2-M10.5, H2-M10.6, H2-M11, H2-M2, H2-M3, H2-M5, H2-M9, H2-Q1, H2-Q10, H2-Q2, H2-Q4, H2-Q6, H2-Q7, H2-T10, H2-T22, H2-T23, H2-T24, H2-T3, Helz2, Herc3, Herc6, Hif1a, I830012O16Rik, Icam1, Ido1, Ifi27, Ifi27l2a, Ifi27l2b, Ifi35, Ifi44, Ifih1, Ifit1, Ifit2, Ifit3, Ifitm1, Ifitm2, Ifitm3, Ifitm5, Ifitm6, Ifitm7, Ifnar2, Il10ra, Il15, Il15ra, Il18bp, Il2rb, Il4ra, Il6, Il7, Irf1, Irf2, Irf4, Irf5, Irf7, Irf8, Irf9, Isg15, Isg20, Isoc1, Itgb1, Itgb2, Itgb7, Jak1, Jak2, Klrk1, Lap3, Lats1, Lats2, Lcp2, Lgals3bp, Ly6e, Lysmd2, March1, March8, Mettl7b, Mt1, Mt2, Mthfd2, Mthfd2l, Mvp, Mx2, Myd88, Nampt, Ncoa2, Ncoa3, Nfkb1, Nfkb2, Nfkbia, Nlrc5, Nmi, Nod1, Nup93, Oas1a, Oas1c, Oas1f, Oas1g, Oas1h, Oas2, Oas3, Oasl1, Oasl2, Ogfr, P2ry14, Parp12, Parp14, Pde4a, Pde4b, Pde4c, Pde4d, Peli1, Peli2, Pfkl, Pfkm, Pfkp, Pim1, Pim3, Pla2g4a, Plscr1, Plscr2, Plscr5, Pml, Pnp, Pnp2, Pnpt1, Psma2, Psma3, Psmb10, Psmb11, Psmb2, Psmb5, Psmb8, Psmb9, Psme1, Psme2, Psme2b, Ptgs1, Ptgs2, Ptpn1, Ptpn11, Ptpn2, Ptpn6, Rapgef6, Rbck1, Ripk1, Ripk2, Rnf213, Rnf31, RP23-322E15.2, Rsad2, Rtp4, Samd9l, Samhd1, Sectm1a, Sectm1b, Selp, Serping1, Slamf7, Slc25a28, Socs1, Socs3, Sod2, Sp110, Sppl2a, Sri, Sspn, St3gal5, St8sia4, Stat1, Stat2, Stat3, Stat4, Tap1, Tapbp, Tdrd7, Tnfaip2, Tnfaip3, Tnfaip6, Tnfsf10, Tor1a, Tor1b, Trafd1, Trim14, Trim21, Trim25, Trim26, Trim31, Trim43a, Trim43b, Trim43c, Txnip, Tyk2, Ube2l6, Upp1, Upp2, Usp18, Vamp5, Vamp8, Vcam1, Wars, Xaf1, Xcl1, Zbp1, Znfx1

Reference

1. Lan Y, Zhang D, Xu C, Hance KW, Marelli B, Qi J, et al. Enhanced preclinical antitumor activity of M7824, a bifunctional fusion protein simultaneously targeting PD-L1 and TGF-β. Sci Transl Med. 2018;10.
